# Real-time direct detection of Criegee intermediates from ozonolysis of alkenes in an atmospheric simulation chamber

**DOI:** 10.1126/sciadv.aeb0618

**Published:** 2026-03-04

**Authors:** Lavinia Onel, Mark A. Blitz, Dwayne E. Heard, Paul W. Seakins, Daniel Stone

**Affiliations:** ^1^ School of Chemistry, University of Leeds, Leeds, UK.; ^2^ National Centre for Atmospheric Science, University of Leeds, Leeds, UK.

## Abstract

Ozonolysis reactions are key oxidation mechanisms for unsaturated volatile organic compounds in the atmosphere, generating Criegee intermediates (CIs) that drive subsequent chemistry. Photolytic laboratory sources of certain CIs have demonstrated unexpectedly high CI reactivity but bypass the complex dynamics that occur in the atmosphere during ozonolysis, which affect yields and reactivity. Here, we report direct measurements of absolute concentrations of the CIs formaldehyde oxide (CH_2_OO) and acetone oxide [(CH_3_)_2_COO] during ozonolysis reactions in an atmospheric simulation chamber made in real-time using UV cavity-enhanced absorption spectroscopy (CEAS). The measurements enable direct determination of stabilized CI yields and reaction kinetics under atmospheric conditions. The technique has the potential to reduce uncertainties associated with ozonolysis chemistry in atmospheric models.

## Teaser

Direct detection of absolute Criegee intermediate concentrations in ozonolysis reactions under atmospheric conditions is reported.

## INTRODUCTION

Ozonolysis reactions, in which ozone initiates the oxidation of unsaturated volatile organic compounds (VOCs) through addition to C═C double bonds, play an important role in the atmosphere as a means of VOC removal and as a source of other reactive species and oxidants including Criegee intermediates (R_2_COO), hydroxyl radicals (OH), and peroxy radicals (HO_2_ and RO_2_) (*1*, *2*). The ozonolysis mechanism and the subsequent chemistry initiated by the species it produces lead to complex cascades of reactions, which affect air quality and climate through the removal of primary pollutants and the production of secondary pollutants and are important in regions affected by both biogenic and anthropogenic emissions (*3*–*6*).

Criegee intermediates are produced in ozonolysis reactions with high internal energy (*1*, *2*) and undergo either rapid unimolecular decomposition or collisional stabilization with surrounding molecules to produce stabilized Criegee intermediates (SCIs) which may also undergo unimolecular decomposition or take part in bimolecular reactions with species such as water vapor, water vapor dimers, and SO_2_ (*7*–*9*). The role of Criegee intermediates in ozonolysis reactions was first suggested in 1949 (*10*), and the potential involvement of SCIs in the atmospheric oxidation of SO_2_ was highlighted in 1971 (*11*–*15*), but challenges associated with direct studies on Criegee intermediate chemistry have hindered our understanding. Developments made possible following the relatively recent discovery of photolytic sources of SCIs for laboratory studies (*16*–*18*), using the flash photolysis technique, have enabled a range of kinetic and spectroscopic studies which have questioned our understanding of the role of SCIs in the atmosphere (*4*, *6*, *19*, *20*). While much progress has been made, the photolytic studies bypass the complex dynamics and collisional stabilization that occur during atmospheric ozonolysis—the natural formation route for SCIs. There are large discrepancies between modeled and inferred SCI abundances in the atmosphere and between the behavior of SCIs observed in photolytic studies and expectations based on observations of stable species in ozonolysis studies, with the kinetics of a number of important SCI processes subject to large uncertainties. For example, reports of rate coefficients for the unimolecular decomposition of the SCI acetone oxide [(CH_3_)_2_COO], which are expected to be independent of pressure under the reported experimental conditions (*21*–*23*), range from (2.7 ± 0.7) s^−1^ (*24*) to (929 ± 220) s^−1^ (*25*) at 298 K, and it has been suggested that differences may be related to the method used to generate the SCI (*26*). There is a limit to the SCIs that can be produced photolytically, with photolytic sources for many atmospherically relevant SCIs yet to be reported and likely to remain a challenge. Moreover, photolytic sources of SCIs provide no details regarding yields of SCIs from ozonolysis reactions.

Observations of the Criegee intermediate formaldehyde oxide (CH_2_OO) have been made during the ozonolysis of ethene using Fourier transform microwave spectroscopy (*27*), and spectroscopic signatures for Criegee intermediates have been noted during the ozonolysis of β-pinene in the infrared region of the spectrum (*28*), but these experiments were not able to determine absolute concentrations or SCI yields. Concentrations of SCIs produced in the ozonolysis of a range of alkenes have been reported from spin trap experiments, with the spin trap technique able to distinguish between several SCIs present within complex mixtures (*29*, *30*). However, no SCI yields or kinetics have been reported using the technique, and there are a number of assumptions made in the analysis of the spin trap signals, and a need for more direct calibration procedures was identified. Cavity ringdown spectroscopy has recently been used in a flow tube experiment to determine absolute concentrations and yields of CH_2_OO in the ozonolysis of ethene at low pressure (<20 torr) (*31*), but thus far, it has not been possible to determine absolute concentrations or yields of SCIs directly in ozonolysis reactions under atmospheric conditions. Yields of SCIs in ozonolysis reactions now adopted in atmospheric models are typically based on indirect measurements making use of SCI scavengers (*9*). At pressures ~1 atm, the SCI yields of CH_2_OO from the ozonolysis of ethene (C_2_H_4_) reported in the literature range from 0.35 (*32*) to 0.59 (*33*), while those for (CH_3_)_2_COO (acetone oxide) from the ozonolysis of tetramethyl ethene [TME; 2,3-dimethylbut-2-ene, (CH_3_)_2_CC(CH_3_)] range from 0.1 (*34*) to 0.65 (*35*). Atmospheric mechanisms typically represent SCI yields by a single value (*36*), with no representation of any pressure dependence, but there is a role for collisional stabilization in SCI formation (*2*, *37*–*39*). The challenges associated with measurements of absolute SCI concentrations in ozonolysis reactions hinder our ability to assess the significance of SCIs in the atmosphere and their impacts on air quality and climate.

We have successfully made direct time-resolved measurements of absolute concentrations of the SCIs CH_2_OO and (CH_3_)_2_COO during ozonolysis reactions in an atmospheric simulation chamber, enabling the determination of yields and reaction kinetics. Ozonolysis reactions of ethene and TME were investigated in the Highly Instrumented Reactor for Atmospheric Chemistry (HIRAC) (*40*) at *T* = 295 K and pressures between 100 and 1000 mbar using a combination of long-path Fourier transform infrared (FTIR) spectroscopy to monitor reactants and stable products, a commercial O_3_ analyzer, and broadband ultraviolet (UV) cavity enhanced absorption spectroscopy (CEAS) to detect the SCIs CH_2_OO and (CH_3_)_2_COO. Full details of the experimental capabilities are given in the Supplementary Materials. Broadband CEAS (330 to 390 nm) facilitates the unambiguous identification of CH_2_OO and (CH_3_)_2_COO in complex reaction mixtures, while the time resolution (100 to 200 ms) and long path length [~228 m at λ ~ 345 nm, where CH_2_OO and (CH_3_)_2_COO have absorption cross sections of >10^−17^ cm^2^ (*9*)] enable measurements of absolute concentrations with the required sensitivity to probe the production and subsequent chemistry of SCIs directly and quantitatively during ozonolysis reactions in real-time.

## RESULTS

### Ozonolysis of ethene

**Fig. 1.**
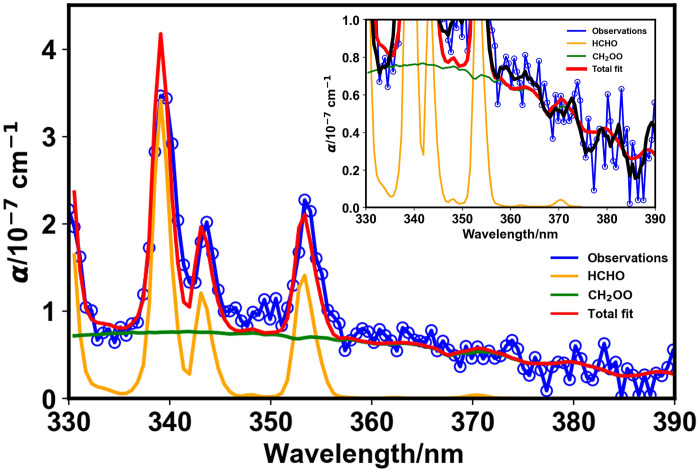
Observed CEAS spectrum (blue), total fit (red), and the individual contributions from the SCI CH_2_OO (green) and HCHO (orange) determined by fitting reference spectra to the measured absorbance. Data shown are from an experiment at 1000 mbar initialized with 2.7 × 10^14^ cm^−3^ O_3_ and 4.4 × 10^15^ cm^−3^ ethene, in the absence of water vapor, averaged between *t* = 3 s and *t* = 7 s following initiation of the ozonolysis reaction by delivery of ethene to the chamber with average concentrations of 5.1 × 10^9^ cm^−3^ CH_2_OO and 6.3 × 10^12^ cm^−3^ HCHO. The inset shows the region of the spectrum in which CH_2_OO exhibits fingerprint vibronic structure in more detail, with the black line showing an averaging kernel applied to the observations along the wavelength axis to show the clear observation of the vibronic structure.

**Fig. 2.**
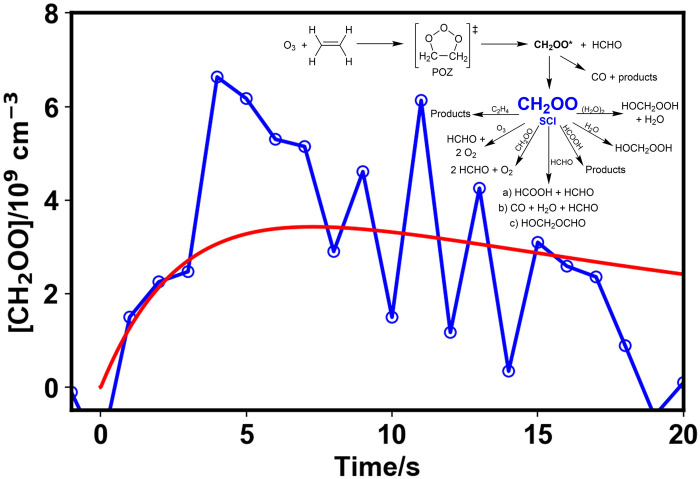
Observed concentrations of stabilized CH_2_OO (blue open circles) and model fit (solid red line) obtained from global fits to observations of CH_2_OO, HCHO, O_3_, and CO from all experiments simultaneously. Data shown are from an experiment at 1000 mbar initialized with 2.7 × 10^14^ cm^−3^ O_3_ and 4.4 × 10^15^ cm^−3^ ethene, in the absence of water vapor. The inset shows the mechanism used to describe the chemistry in the system (also shown in fig. S6). POZ, primary ozonide.

Figure 1 shows CEAS measurements made during the ozonolysis of ethene, demonstrating the presence of the SCI CH_2_OO and formaldehyde (HCHO), with each clearly identifiable by virtue of their unique vibronic structures which provide unmistakable fingerprints in the UV spectrum. Concentrations of CH_2_OO obtained by fitting reference spectra (*41*, *42*) to the CEAS measurements are shown in Fig. 2. Measurements of HCHO, made by both CEAS and FTIR spectroscopy, ozone, and other species made by FTIR spectroscopy are provided in the Supplementary Materials.

Observations reveal production of the SCI within several seconds of mixing ethene with ozone present in the chamber, followed by decay on a timescale of seconds to tens of seconds. Experiments performed over a range of relative humidities (RH; 0 to 20% at 295 K, all at 1000 mbar) demonstrated the suppression of the SCI signal at the higher RH values investigated owing to the rapid reaction of CH_2_OO with water dimers [(H_2_O)_2_] (*43*, *44*), with experiments under such conditions leading to low CH_2_OO concentrations.

Detailed analysis was performed using a model which considered the production, stabilization, and subsequent chemistry of CH_2_OO, as well as effects of mixing in the reaction chamber (a summary of the mechanism is shown in Fig. 2, and a full description is given in the Supplementary Materials). At low RH, the loss of CH_2_OO in the chamber was dominated by its reaction with HCHO. Global fits to observations of CH_2_OO, HCHO, O_3_, and CO were performed to assess the SCI yield, the kinetics of CH_2_OO + HCHO, and the yield of HCHO regenerated in the reaction between CH_2_OO and HCHO, which is the subject of some uncertainty in the literature. Results are shown in Fig. 2. The fits gave an SCI yield of (0.38 ± 0.09) at 1000 mbar, with all uncertainties reported for this work at the 1σ level. The SCI yield compares well to the current IUPAC recommendation (*9*) of (0.42 ± 0.10) at 1000 mbar based on previous indirect measurements which range between 0.35 (*32*) and 0.59 (*33*) at room temperature and atmospheric pressure.

The rate coefficient obtained for CH_2_OO + HCHO was (6.0 ± 1.9) × 10^−12^ cm^3^ s^−1^, with the reaction representing 70% of the total SCI loss in the absence of water vapor. Previous experimental work using photolytic precursors to CH_2_OO has reported rate coefficients of (4.11 ± 0.25) × 10^−12^ cm^3^ s^−1^ (*45*) and (3.50 ± 0.35) × 10^−12^ cm^3^ s^−1^ (*46*). Theory indicates that the reaction between CH_2_OO and HCHO proceeds initially through a secondary ozonide (SOZ) (*47*–*49*), which decomposes rapidly to produce HCOOH + HCHO or CO + H_2_O + HCHO, either directly (*47*, *48*) or through the formation of the intermediate hydroxymethyl formate (HMF; HOCH_2_OCHO) (*47*). The calculations thus suggest the potential for regeneration of HCHO but differ on the significance of the two decomposition channels and uncertainty regarding the potential role of stabilization of the SOZ or HMF. Experimental measurements of the products of CH_2_OO + HCHO have been made by Luo *et al.* (*45*) at pressures between 15 and 60 torr in flash photolysis experiments using infrared frequency comb spectroscopy, with results indicating a yield of ~43% for HCOOH and ~57% for CO and suggesting 100% regeneration of HCHO. However, the experiments reported by Luo *et al.* were performed at relatively low pressures, and the stabilization of both the SOZ and HMF will be pressure dependent. The SOZ has been detected at atmospheric pressure using Fourier transform microwave spectroscopy, although it was not possible to determine the yield (*27*) and HMF has been reported as one of the products at atmospheric pressure (*27*). Previous chamber studies of ethene ozonolysis at atmospheric pressure have also reported production of HMF from the reaction of CH_2_OO with HCHO (*32*, *50*–*53*), although the spectral assignment of HMF has been questioned and there is the possibility that the infrared peaks attributed to HMF result from products of the reaction between CH_2_OO and HCOOH (*54*).

The best fit to observations made in this work gave a regeneration yield for HCHO from CH_2_OO + HCHO of (23 ± 9)%, indicating substantial stabilization of the SOZ or HMF under the conditions used in this work. FTIR measurements made in this work provide evidence for production of HCOOH and CO, but HCOOH could not be accurately quantified and observations of CO could be explained by a number of potential sources (further details are given in the Supplementary Materials). Modeled profiles for HCOOH followed observations closely (see the Supplementary Materials), suggesting that the HCOOH observed was predominantly formed via the reaction between CH_2_OO and HCHO. The reaction between CH_2_OO and HCOOH has been demonstrated to be rapid [*k* = ($1.1_{-0.2}^{+0.3}$) × 10^−10^ cm^3^ s^−1^ at 298 K] (*9*, *55*–*57*), and its potential impact thus requires consideration. Although the yield of HCOOH from the reaction between CH_2_OO and HCHO could not be assigned accurately, assuming that 100% of the coproduct of the HCHO regenerated is HCOOH gives an upper limit of 23%. At a yield of 23% for production of HCOOH from CH_2_OO + HCHO, the model used to fit the data indicates that the reaction between CH_2_OO and HCOOH represents an important removal mechanism for CH_2_OO (14% of the total loss at 0% RH), but there is minimal sensitivity of the SCI yield and kinetics of CH_2_OO + HCHO to the yield of HCOOH assumed in the model (see the Supplementary Materials). Results for the SCI yield and kinetics of CH_2_OO + HCHO reported in this work are given for the yield of HCOOH of 23% but incorporate uncertainties associated with the model sensitivity to HCOOH.

Following the observed decay of CH_2_OO, growth of an unidentified product species was observed in the CEAS data. The product signal was suppressed in the experiments performed at the highest RH, indicating its production following chemistry of CH_2_OO occurring in competition with the reaction between CH_2_OO and water. The time dependence of the signal suggests a link to the modeled products of CH_2_OO + HCHO or CH_2_OO + HCOOH, which could not be distinguished owing to the production of HCOOH from CH_2_OO + HCHO and the rapid subsequent reaction of CH_2_OO with HCOOH, and we speculate that the absorption results from species generated from chemistry of the SOZ or HMF formed via CH_2_OO + HCHO or oligomer formation resulting from CH_2_OO + HCOOH. Further details are discussed in the Supplementary Materials.

The high yield of HCHO from ethene ozonolysis and the dominance of CH_2_OO + HCHO and subsequent chemistry, combined with the relatively slow reactivity of ethene toward ozone, make observations of CH_2_OO in ethene ozonolysis particularly demanding. The CEAS technique enables quantitative measurements and a means to assess the SCI yield and reaction kinetics directly.

### Ozonolysis of TME

In comparison to ethene, TME has both higher reactivity toward ozone and lower yields of HCHO relative to the SCI (*9*), reducing the potential impact of SCI + HCHO and subsequent reactions and leading to the production of higher SCI concentrations with longer lifetimes. TME ozonolysis thus offers the potential for more detailed analysis of SCI yields and chemistry.

**Fig. 3.**
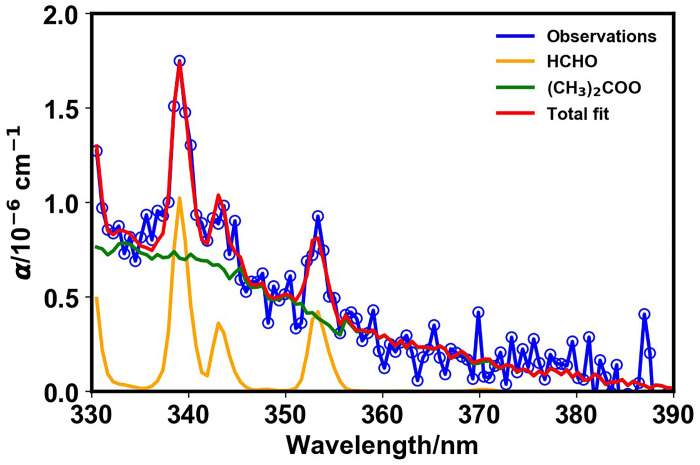
Observed CEAS spectrum (blue), total fit (red), and the individual contributions from the SCI (CH_3_)_2_COO (green) and HCHO (orange) determined by fitting reference spectra to the measured absorbance. Data shown are from an experiment at 1000 mbar initialized with 7.1 × 10^13^ cm^−3^ O_3_ and 1.4 × 10^14^ cm^−3^ TME, at *t* = 6 s following initiation of the ozonolysis reaction by delivery of TME to the chamber. The fits gave [(CH_3_)_2_COO] = 4.1 × 10^10^ cm^−3^ and [HCHO] = 2.0 × 10^12^ cm^−3^.

**Fig. 4.**
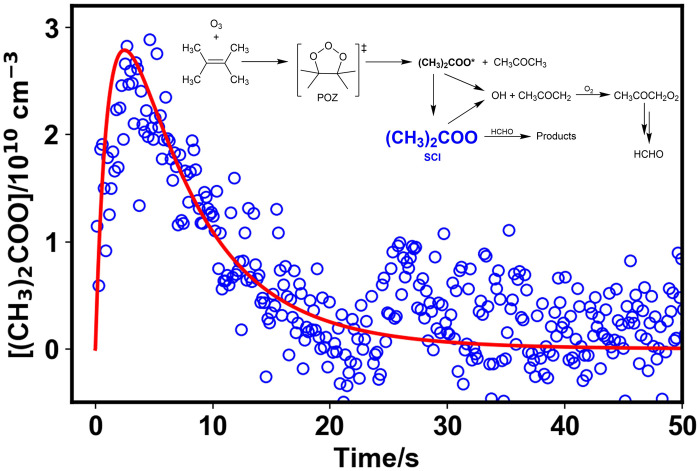
Observed concentrations of stabilized (CH_3_)_2_COO (blue open circles) and model fit (solid red line) obtained from global fits to observations of (CH_3_)_2_COO, HCHO, acetone, and O_3_ from all experiments simultaneously. Data shown are from an experiment at 100 mbar initialized with 2.0 × 10^14^ cm^−3^ O_3_ and 1.3 × 10^14^ cm^−3^ TME. The inset shows the mechanism used to describe the chemistry in the system (also shown in fig. S10).

Figure 3 shows a typical CEAS spectrum recorded during the ozonolysis of TME, demonstrating the production of the SCI (CH_3_)_2_COO and formaldehyde, which is produced by chemistry of the peroxy radical CH_3_COCH_2_O_2_ generated via decomposition of the Criegee intermediate. Concentration-time profiles for (CH_3_)_2_COO and HCHO determined by fitting to the CEAS measurements are shown in Fig. 4. Observations of ozone and acetone made via FTIR spectroscopy are discussed in the Supplementary Materials. Features in the CEAS data could be explained by the presence of (CH_3_)_2_COO and HCHO alone. The SCI displayed rapid growth, on the timescales of seconds, followed by removal on timescales of tens of seconds. Formaldehyde displayed growth to maximum, and relatively constant, concentrations on timescales of tens of seconds. Experiments were performed over a wide range of pressures (100 to 1000 mbar) and initial concentrations (details are given in the Supplementary Materials) to vary the impacts of collisional stabilization of the nascent excited Criegee intermediate, to decouple the kinetics of SCI production and loss, and to provide sensitivity to the competition between removal via unimolecular decomposition and other potential bimolecular reactions. Global fitting to the observations for (CH_3_)_2_COO, HCHO, acetone, and O_3_ from experiments conducted over a range of initial concentrations and pressures enabled simultaneous determination of pressure-dependent SCI yields and kinetics of SCI reactions. Full details of the model are given in the Supplementary Materials.

Figure 4 compares the observations and model performance for (CH_3_)_2_COO and HCHO (fits to O_3_ and acetone are given in the Supplementary Materials). The fit results indicate an SCI yield of (0.45 ± 0.09) at *p* = 100 mbar and (0.61 ± 0.18) at *p* = 1000 mbar. Previous indirect measurements have indicated pressure dependence in the yield (*37*, *38*) but range between 0.1 (*34*) and 0.65 (*35*) at atmospheric pressure. The current IUPAC recommendation (*9*) for the yield of the SCI from TME ozonolysis is (0.38 ± 0.10) at 298 K and 1000 mbar, although the MCM (*36*), which is typically used as a benchmark for atmospheric chemical modeling, adopts a yield of unity that does not vary with pressure. Indirect methods used to determine SCI yields in previous work are often reliant on the use of SCI scavengers (*9*, *58*), requiring the loss of the SCI via reaction with the scavenger to be considerably faster than other loss processes, which may not have always been the case.

The dominant loss process for the SCI (CH_3_)_2_COO in this work was unimolecular decomposition. The fit to the observed SCI concentrations gave a decomposition rate coefficient of (156 ± 68) s^−1^, with no significant effects of pressure observed within the fit uncertainties. Indirect measurements based on observations of OH production in TME ozonolysis have suggested rate coefficients for SCI decomposition of (2.7 ± 0.7) s^−1^ at 10 torr and (6.4 ± 0.9) s^−1^ at 100 torr (*24*). Studies using photolytic precursors to (CH_3_)_2_COO have reported room temperature rate coefficients for decomposition varying between (305 ± 70) s^−1^ (*23*) and (899 ± 42) to s^−1^ (*26*). Relative rate studies of SCI reactions in TME ozonolysis, involving observation of SO_2_ removal or H_2_SO_4_ production resulting from the reaction between the SCI and SO_2_, have indicated room temperature rate coefficients between (605 ± 109) s^−1^ (*59*) and (929 ± 220) s^−1^ (*25*). Despite differences in reported kinetics, the previous absolute studies of (CH_3_)_2_COO decomposition have been in agreement that the reaction is at, or near to, the high pressure limit at pressures above 10 torr (*21*–*23*), and a role for quantum tunneling in the reaction has been demonstrated under ambient conditions which minimizes the effects of pressure (*60*–*62*).

Observations made in this work also indicate a reaction of (CH_3_)_2_COO with HCHO. The fit to the data indicates a mean rate coefficient of (9.7 ± 6.8) × 10^−13^ cm^3^ s^−1^ for the reaction between (CH_3_)_2_COO and HCHO over the pressure range investigated, which is similar to those for CH_2_OO + HCHO and other SCI + aldehyde reactions reported in the literature (*45*, *46*). For experiments at 1000 mbar, decomposition of the SCI represents 88% of the total SCI loss and reaction with HCHO represents 12% of the loss.

## DISCUSSION

Results reported in this work represent direct measurements of absolute SCI concentrations in ozonolysis reactions under atmospheric conditions, providing opportunities to study SCI yields and kinetics. There is broad potential for application of CEAS to further studies of SCI yields in ozonolysis reactions, which to date have only been possible via indirect methods, and of Criegee intermediate chemistry, particularly where photolytic sources are not available. CEAS offers both specificity and sensitivity and the potential for determination of conformer-dependent SCI yields which are now lacking in atmospheric models but can have potentially important impacts where SCI conformers display differences in reactivity and product yields (*18*). Although absolute cross sections are required for quantitative determination of SCI yields, it has been shown that calculated cross sections can accurately reproduce experimental values (*63*–*66*). Direct measurements of SCI yields and kinetics will provide further constraints to the development of the complex reaction mechanisms used to model atmospheric chemistry and composition and thus to understand and predict air quality and climate.

## MATERIALS AND METHODS

Experiments described in this work were performed in the HIRAC at 100 and 1000 mbar of synthetic air obtained by mixing high purity O_2_ (BOC, >99.999%) and N_2_ (BOC, >99.998%). The chamber was evacuated between experiments and could be pumped from ambient pressure to ~2.5 × 10^−3^ mbar within ~70 min. Pressures were measured using a capacitance manometer (Leybold Ceravac CTR90) and a Pirani gauge (Leybold Thermovac TTR91) and controlled by a roots blower (Leybold Ruvac WAU251) backed by a rotary pump (Leybold Trivac D40B), with a charcoal filled catchpot trap (BOC Edwards ITC300). All experiments reported here were conducted at room temperature (~295 K).

Ozone was delivered to the chamber by passing a flow of high purity O_2_ (BOC, >99.999%) through a commercial ozone generator (Fischer Technology OZ500) and into the chamber until the desired concentration, measured by a commercial ozone analyzer (Thermo Electron Environmental Instruments, model 49C), had been reached. Following the generation of ozone, ethene (Air Products, 99.5%) or TME (Sigma-Aldrich, ≥99%) was prepared in a 1-liter stainless steel mixing vessel and flushed into HIRAC with a stream of nitrogen to initiate the chemistry within the chamber. Water vapor was delivered to the chamber by injecting deionized liquid water in a stream of N_2_.

For experiments with ethene, all experiments were performed with a chamber pressure of 1000 mbar. Initial reagent concentrations were (4.4 to 12.1) × 10^15^ cm^−3^ for ethene, (1.5 to 3.2) × 10^14^ cm^−3^ for ozone, and (0 to 1.3) × 10^17^ cm^−3^ for water vapor (RH, 0 to 20%). For experiments with TME, experiments were performed at chamber pressures of 100 or 1000 mbar. At 100 mbar, initial reagent concentrations were (0.8 to 2.3) × 10^14^ cm^−3^ for TME and (0.6 to 4.3) × 10^14^ cm^−3^ for ozone. At 1000 mbar, initial reagent concentrations were (0.2 to 3.4) × 10^14^ cm^−3^ for TME and (0.5 to 7.1) × 10^14^ cm^−3^ for ozone.

HIRAC was equipped with an in situ multipass FTIR spectrometer (Bruker IFS66) which used a modified Chernin cell aligned along the long-axis of the chamber to give a total path length of ~128.5 m. IR spectra were recorded every 30 to 60 s as averages of 30 to 100 scans at a spectral resolution of 1 cm^−1^.

Broadband UV CEAS was used to monitor species absorbing in the region of 330 of 390 nm. The cavity was formed by a pair of highly reflective mirrors (25.0 mm in diameter; radius of curvature, 1000 mm; LAYERTEC 152456 batch D219A045) with a reflectivity of >99.2% and a transmission of ~0.2% in the range of 330 to 390 nm. The mirrors were mounted across the short-axis of the chamber in custom-built mounts that allow fine alignment of the mirrors while maintaining a gas-tight seal, giving a distance of 1.4 m between the mirrors and a total absorption path length of ~280 m at 345 nm (see the Supplementary Materials for details of the path length measurement).

CEAS measurements were made using a laser-driven light source (LDLS; Energetiq EQ-99X), which provided ~10 mW cm^−2^ of light at wavelengths between 200 and 800 nm with near constant radiance across the spectral range. The LDLS output was collimated by a UV-enhanced Al off-axis parabolic mirror (2″; Thorlabs MDP229-F01) and aligned into the optical cavity by a UV-enhanced Al mirror (1″; Thorlabs PF10-03-F01). Light exiting the cavity was directed by a second UV-enhanced Al mirror through a 1-cm quartz cuvette containing acetone to reduce the impacts of light outside the spectral range of the cavity and focused onto a fiber optic by a fiber launcher (Elliot Scientific MDE122). Output from the fiber was directed onto a spectrograph (CP140-103 Imaging Spectrograph, Horiba), giving spectral resolution of 1.5 nm full width at half maximum, and imaged onto a line-scan charge-coupled device detector (S7030-1006 FFT, Hamamatsu) which has been used in our previous work. Intensity spectra were recorded at intervals of 100 to 200 ms, with intensity measurements started ~60 s before any chemistry was initiated in the chamber to provide a measure of the background intensity of the light. Wavelength calibration was achieved through measurements of the well-known Hg emission spectrum from a low-pressure Hg pen-ray lamp (Oriel).

## Supplementary Materials

### The PDF file includes:

Supplementary Text Figs. S1 to S12 Tables S1 to S7 Legend for data S1 References
