# Supplementary material for: Real-time direct detection of Criegee intermediates from ozonolysis of alkenes in an atmospheric simulation chamber
Source: Sci Adv. 2026 Mar 4;12(10):eaeb0618. doi: 10.1126/sciadv.aeb0618 (PMC12959382; doi:10.1126/sciadv.aeb0618)
Supplement: Supplementary file 1 — Supplementary Text Figs. S1 to S12 Tables S1 to S7 Legend for data S1 References [file sciadv.aeb0618_sm.pdf]

Science Advances
AAAS

Supplementary Materials for

Real-time direct detection of Criegee intermediates from ozonolysis of alkenes in an atmospheric simulation chamber

Lavinia Onel *et al.*

Corresponding author: Daniel Stone, d.stone@leeds.ac.uk

Sci. Adv. **12**, eaeb0618 (2026)
DOI: 10.1126/sciadv.aeb0618

The PDF file includes:

Supplementary Text
Figs. S1 to S12
Tables S1 to S7
Legend for data S1
References

Other Supplementary Material for this manuscript includes the following:

Data S1

Supplementary Text

1. HIRAC

Experiments were performed in the Highly Instrumented Reactor for Atmospheric Chemistry (HIRAC), which has been described in detail in previous work.(*40, 68*) A schematic is shown in Figure S1. HIRAC is a cylindrical stainless steel atmospheric simulation chamber with an internal volume of ~2.25 m^3 and surface to volume ratio of ~5.8 m^{-1}. Four circulation fans mounted in pairs at each end of the chamber ensure homogeneous mixing (see Section S4).

2. Determination of the CEAS total absorption path length

The absorption path length of the CEAS system was determined by measuring the absorbance of known concentrations of NO_2 (Sigma-Aldrich, 99.5 %) prepared manometrically. Figure S2 shows the measured absorbance by CEAS, determined via the Beer-Lambert law (see Section S3), and the total absorption path length for the CEAS measurements, determined from the measured absorbance, the known concentration of NO_2, and reference absorption cross-sections for NO_2.(*42*) The highly reflective mirrors used to generate the optical cavity have wavelength-dependent reflectivity, varying from ~99.2 % at ~373 nm to ~99.7 % at ~343 nm, resulting in wavelength-dependent total absorption path length that varies from ~228 m at ~345 nm to ~100 m at 375 nm, as shown in Figure S2.

3. Determination of concentrations for species observed by absorption spectroscopy

3.1 CEAS measurements

Cavity enhanced absorbance spectra were determined from measured intensity data and related to the concentration of each species present using the Beer-Lambert law (Equation S1):

$$A_{\lambda,t} = \ln\left(\frac{I_{\lambda,0}}{I_{\lambda,t}}\right) = \sum_i \sigma_{i,\lambda} c_{i,t} l_\lambda \qquad \text{(Equation S1)}$$

where $A_{\lambda,t}$ is the total absorbance at wavelength λ and time t, $I_{\lambda,0}$ is the average pre-reaction light intensity at wavelength λ, $I_{\lambda,t}$ is the light intensity at wavelength λ and time t, $\sigma_{i,\lambda}$ is absorption cross-section of species i at wavelength λ, $c_{i,t}$ is the concentration of species i at time t, and l_λ is the absorption path length at wavelength λ (see Section S2). For experiments involving ethene, intensity data were averaged on to 1 s timescales. For experiments involving TME, intensity data were analysed as recorded (i.e. on timescales of 100 to 200 ms).

In order to account for the wavelength-dependent absorption path length, data were analysed in terms of the absorption coefficients, $\alpha_{\lambda,t}$, given by the ratio $A_{\lambda,t}/l_\lambda$. Concentrations of species observed by broadband UV CEAS (i.e. CH_2OO or $(CH_3)_2COO$ and HCHO) were determined by least-squares fitting of reference absorption cross-sections,(*21, 41, 42, 67*) $\sigma_{i,\lambda}$, to the measured absorption coefficients, $\alpha_{\lambda,t}$, at each time point. Example fits to spectra and concentration-time profiles are shown in the main text (Figures 1-4), and in Figures S7 and S11 (Sections S5 and S6) with results obtained by numerical modelling of the reaction systems.

Absorption cross-sections for CH_2OO used in the fits were taken from our previous work,(*41*) which were in good agreement with measurements reported in other work (*69, 70*) and have an uncertainty of ~21 %. Cross-sections for $(CH_3)_2COO$ were taken from measurements of the $(CH_3)_2COO$ spectrum determined in flash photolysis experiments using $(CH_3)_2CI_2$ (*21*) and scaled to the absolute cross-section determined at 308.4 nm in photodepletion experiments,(*67*) which has an uncertainty of ~8 %. Uncertainties in the cross-sections are the main source of uncertainty in the concentrations determined for the Criegee intermediates.

3.2 FT-IR measurements

In order to account for effects of instrument resolution and pressure-broadening of spectra, reference FT-IR spectra were recorded by delivering known concentrations of the reference compound to HIRAC under the same conditions as those used in ozonolysis experiments. Concentrations of species observed by FT-IR spectroscopy during ozonolysis reactions were subsequently determined by fitting the reference spectra to the observed spectra using the Beer-Lambert law (Equation S1). Figure S3 shows example fits to spectra. Concentration-time profiles determined by fitting to the spectra are given in Sections S5 and S6 with results obtained by numerical modelling of the reaction systems.

3.3 Comparison of CEAS and FT-IR measurements

Figure S4 shows a typical comparison of the HCHO concentration-time profile determined by CEAS to that determined by FT-IR spectroscopy. In general, there was good agreement between the two methods.

4. Mixing effects in HIRAC

Since the ozonolysis reactions studied were initiated by injection of the alkene into HIRAC and the reactions occur on relatively short timescales, understanding the mixing time is critical for extracting quantitative data.

Effects of mixing in HIRAC were evaluated by delivering NO_2 or HCHO to the chamber at either 100 or 1000 mbar using the same delivery port close to the optical cavity used to deliver ethene or TME.

Figure S5 shows the concentration-time profiles for NO_2 and HCHO measured using CEAS. At each pressure the concentration-time profiles for NO_2 and HCHO display the same shape, indicating similar mixing effects for the two species. At 100 mbar the concentration in the optical cavity increased gradually to a constant level in ~20 s, at which point the gas mixture in the chamber was homogeneous. At 1000 mbar the concentration of the compound delivered to the CEAS optical cavity rose to a maximum value within 3 to 4 s and then decreased to a constant concentration in ~15 s, at which point the gas mixture in the chamber was homogeneous.

The effects of mixing were described by the mechanism given below:

$$\mathrm{X_{del}} \xrightarrow{k_{\mathrm{del}}} \mathrm{X_{inside\ cavity}} \underset{k_{\mathrm{mix}}}{\overset{k_{\mathrm{mix}}}{\rightleftharpoons}} \mathrm{X_{outside\ cavity}}$$

Delivery of the compound to the optical cavity was approximated by a first-order process, with rate coefficient k_{del}, followed by mixing of the compound between the region probed by the optical cavity and the region outside the cavity until equilibrium was reached. Movement of species between the two regions was approximated by the first-order rate coefficient k_{mix}.

The concentration-time profiles shown in Figure S5 were fit at each pressure to find the solution to the rate equations for the processes shown in Scheme 1, which is given by Equation S2:

$$[\mathrm{X}]_t = \frac{2\,[\mathrm{X}]_{\mathrm{eq}}(k_{\mathrm{del}} - k_{\mathrm{mix}})}{(2k_{\mathrm{mix}} - k_{\mathrm{del}})}\{\exp(-k_{\mathrm{del}}t) - \exp(-2k_{\mathrm{mix}}t)\} + [\mathrm{X}]_{\mathrm{eq}}\{1 - \exp(-2k_{\mathrm{mix}}t)\} \qquad \text{Equation S2}$$

The parameters $[X]_{eq}$ and k_{del} were treated as local parameters for each concentration-time profile, while k_{mix} was treated as a global parameter and shared by all the measurements carried out at a particular pressure. The fits gave $k_{mix} = (0.568 \pm 0.053)\ s^{-1}$ at 100 mbar and $k_{mix} = (0.129 \pm 0.002)\ s^{-1}$ at 1000 mbar, where errors are statistical uncertainties at the 1σ level.

In all ozonolysis experiments, O_3 was prepared in the chamber and mixed before the alkene was delivered, with chemistry initiated by the delivery of the alkene to the chamber. Numerical models used to describe the ozonolysis of C_2H_4 and TME (Sections S5 and S6) used the description of delivery and mixing shown in Scheme 1 for C_2H_4 and TME, with k_{del} treated as a local parameter in the model. For all other species in the models, k_{mix} was used to describe the movement of species between the region probed by the cavity and the region outside the cavity. For all species, k_{mix} = (0.568 ± 0.053) s^{-1} at 100 mbar and k_{mix} = (0.129 ± 0.002) s^{-1} at 1000 mbar.

5. Ozonolysis of ethene

5.1 Initial conditions

Table S1 summarises the initial conditions used in experiments to investigate the ozonolysis of ethene. Experiments were performed at a total pressure of 1000 mbar with ethene in excess of ozone for a range of relative humidities (0–20 %) in order to vary the loss rate of stabilised CH_2OO whilst maintaining the SCI yield at a constant value.

5.2 Model analysis

The ozonolysis of ethene was investigated using a model developed in the ChemPy and SciPy packages in Python, with mixing in HIRAC represented in the model as described in Section S4. The mechanism used in the model is given in Table S2 and summarised in Figure S6.

The reaction between O_3 and ethene was assumed to produce HCHO with unity yield and the SCI CH_2OO, with the rate coefficient for the reaction constrained to the current IUPAC recommendation.(*9*) Radical species produced by decomposition of the excited Criegee intermediate are expected to react primarily with ethene, or peroxy radicals formed through oxidation of ethene, and were not found to impact the modelled behaviour of the SCI. The use of excess ethene over O_3 ensured that O_3 was the limiting reagent for SCI production and that additional losses of ethene resulting from the presence of radical species did not significantly impact the determination of the SCI yield. Radical species were thus excluded from the model.

Rate coefficients for reactions of stabilised CH_2OO with O_3, ethene, HCOOH, and water monomers (H_2O) and dimers ($(H_2O)_2$) were constrained to values reported in the literature from experiments using flash photolysis of CH_2I_2 to generate the Criegee intermediate (see Table S2). The self-reaction of CH_2OO was included in the model, with kinetics constrained to previous measurements, but the reaction was not significant under the conditions employed in this work. Decomposition of stabilised CH_2OO was not included in the model owing to the slow reaction (<< 1 s^{-1} at 1000 mbar and 298 K).(*71*)

Modelled concentrations of CH_2OO, HCHO, O_3, and CO were fit globally to observations made by CEAS, for CH_2OO and HCHO, the commercial analyser, for O_3, and FT-IR, for CO, for all experiments listed in Table S1 to investigate the SCI yield and the impact of the reaction between CH_2OO and HCHO. The model was initialised with concentrations of ozone, ethene, and, where relevant, water vapour (monomers and dimers). Fits to observations of CH_2OO, HCHO, O_3, and CO from all experiments listed in Table S1 were made simultaneously, with the fit parameters treated globally and shared between all datasets. Observations and modelled concentrations of each species used in the fits were normalised by dividing by the order of magnitude of the concentration of that species. For example, concentrations of CH_2OO, which were on the order of 10^9 cm^{-3}, were divided by 10^9, and concentrations of HCHO, which were on the order of 10^{14} cm^{-3}, were divided by 10^{14}, such that all values used in the fit were between 0 and 10. Normalisation of the concentrations acted to weight the data in order to avoid potential biases in the fits owing to differences between the concentrations of different species of several orders of magnitude.

The fit results are summarised in Table S2, with example fits shown in Figure S7. The best fit to the observations gave an SCI yield of (0.38 ± 0.09), a rate coefficient for reaction between CH_2OO and HCHO of (6.0 ± 1.9) × 10^{-12} cm^3 s^{-1}, and a regeneration yield of HCHO of (23 ± 9) % from the reaction between CH_2OO and HCHO. Results presented in this work assume an upper limit of 23 % for production of HCOOH from the reaction of CH_2OO + HCHO, the impact of which is discussed further in Section 5.4.

Figure S8 shows the relative contributions of the SCI reactions in the model to the total SCI loss, using the results for the SCI yield and kinetics determined in this work, for an experiment in the absence of water vapour. The reaction between the SCI and HCHO was the dominant loss for the SCI in the model, representing 70 % of the total SCI loss, followed by reaction of the SCI with HCOOH, which represented a further 14 % of the total loss. Reactions with O_3 and ethene represented 11 % and 5 % of the SCI loss, respectively, with SCI self-reaction representing < 1 % of the total loss. For the experiment performed at the highest relative humidity employed in this study, the reactions of the SCI with water monomers and dimers represented 4 % and 18 % of the total SCI loss, with remaining losses resulting from reactions with HCHO (57 %), HCOOH (11 %), O_3 (7 %), C_2H_4 (3 %), and self-reaction (< 1 %).

5.3 SCI Yields

Table S3 summarises previous measurements of SCI yields in the ozonolysis of ethene. Results obtained in this work indicate a yield of (0.38 ± 0.09), assuming that the reaction between CH_2OO and HCHO produces HCOOH with a yield of 23 %, which is discussed further in Section 5.4. Results are in good agreement with the current IUPAC recommendation (*9*) of (0.42 ± 0.10) at 1000 mbar and with the value of 0.37 currently adopted in the Master Chemical Mechanism (MCM),(*36*) which is used worldwide as a benchmark for atmospheric modelling.

5.4 SCI + HCHO Kinetics

The rate coefficient for reaction between CH_2OO and HCHO was determined in this work to be (6.0 ± 1.9) × 10^{-12} cm^3, compared to previous measurements of (4.1 ± 0.5) × 10^{-12} cm^3 s^{-1} (*45*) and (3.50 ± 0.35) × 10^{-12} cm^3 s^{-1} (*46*) obtained in flash photolysis experiments.

Products of CH_2OO + HCHO were also investigated in one of the photolytic studies,(*45*) which indicated two main product channels at pressures between 15 and 60 Torr, leading to formation of formic acid (HCOOH, ~43 % yield) and CO + H_2O (~57 % yield), both of which are expected to regenerate HCHO as a co-product. Calculations of the potential energy surface (*47-49*) for the reaction indicate that it proceeds via initial production of a secondary ozonide which is expected to decompose rapidly to produce HCOOH + HCHO or CO + H_2O + HCHO,(*47, 48*) either directly or via production of the intermediate hydroxymethyl formate ($HOCH_2OCHO$, HMF). The calculations thus also indicate regeneration of HCHO, but disagree in the relative significance of channels producing HCOOH + HCHO or CO + H_2O + HCHO,(*47, 48*) and the potential sensitivity of the calculations to collisional energy transfer processes has been noted.(*47*) Evidence for production of HMF (*32, 50-53*) and for stabilisation of the secondary ozonide (*27*) has been reported in experiments performed at atmospheric pressure, although it was not possible to determine the yield.(*27*) Observations of HMF have also been reported in chamber studies at atmospheric pressure, which were attributed to reaction of CH_2OO with HCHO,(*32, 50-53*) although the possibility of interferences in spectral assignments of HMF production CH_2OO + HCHO owing to products generated from the reaction of CH_2OO with HCOOH in chamber studies at atmospheric pressure has also been discussed.(*54*)

Results obtained in this work indicate that the yield of HCHO regenerated in the reaction between CH_2OO and HCHO is (23 ± 9) %. The observed HCHO regeneration yield thus indicates that there is significant stabilisation of an addition product formed between CH_2OO and HCHO at 1000 mbar.

Production of HCOOH was observed via FT-IR spectroscopy, as shown in Figure S3, which was likely produced in the reaction channel CH_2OO + HCHO → HCHO + HCOOH but it was not possible to determine absolute concentrations owing to overlapping spectral features and calibration uncertainties. Figure S7 shows the time profiles for the observed HCOOH signal and modelled concentrations of HCOOH normalised to the maximum values, with the agreement suggesting that the HCOOH observed was formed via the reaction between CH_2OO and HCHO.

Production of CO was also observed by FT-IR spectroscopy, and the observations could be equally well reproduced by the model in fits which assume production from only the initial reaction between O_3 and ethene, those which assume production from only CH_2OO + HCHO, and those which assume production from a combination of both reactions. It was thus not possible to assign the sources of CO with confidence, but differences in the method of production of CO assumed in the model did not impact the modelled SCI behaviour.

The yield of HCOOH from the reaction between CH_2OO and HCHO could thus not be assigned accurately, but has an upper limit of 23 %, assuming that 100 % of the co-product of the HCHO regenerated is HCOOH. Given the rapid reaction between CH_2OO and HCOOH (($1.1^{+0.3}_{-0.2}$) × 10^{-10} cm^3 s^{-1},(*9, 55-57*) the sensitivity of the model fits to the yield of HCOOH from CH_2OO + HCHO assumed in the model was investigated by comparing fit results from a model in which the yield of HCOOH from CH_2OO + HCHO was assumed to be 23 % to those from a model in which the yield was assumed to be zero.

The fits show little sensitivity of the SCI yield and kinetics of CH_2OO + HCHO to the yield of HCOOH assumed in the model. Compared to the model fits which used a yield of 23 % HCOOH and gave an SCI yield of (0.38 ± 0.09) and rate coefficient for the reaction between CH_2OO and HCHO of (6.0 ± 1.9) × 10^{-12} cm^3 s^{-1}, model fits using a yield of HCOOH of zero gave an SCI yield of (0.39 ± 0.07) and a rate coefficient for CH_2OO + HCHO of (7.3 ± 2.8) × 10^{-12} cm^3 s^{-1}. Results for the SCI yield and kinetics of CH_2OO + HCHO reported in this work are given for the yield of HCOOH of 23 %, but incorporate uncertainties associated with the model sensitivity to HCOOH. Investigation of product yields from the reaction between CH_2OO and HCHO as a function of pressure warrants further investigation.

5.5 CEAS observations of product formation

Following the observed decay of CH_2OO, the CEAS spectra show the growth of an unidentified product species that was inhibited in experiments in the presence of water vapour, and thus appeared to result from chemistry of the SCI occurring in competition with the reaction between the SCI and water. Figure S9 shows observations of the product species made in experiments performed over a range of relative humidities.

The time dependence and suppression of the signal in the presence of water vapour suggests a link between the observed product species and the products of CH_2OO reactions with HCHO or HCOOH, which dominate the chemistry in the system and impact CH_2OO over longer timescales than reactions with other species (see Figure S8). However, the role of products formed from reactions of CH_2OO with HCHO or HCOOH could not be distinguished owing to the production of HCOOH from CH_2OO + HCHO and the rapid subsequent reaction of CH_2OO with HCOOH. Comparison of the observed product signal in the absence of water vapour and that observed in experiments at the highest RH investigated indicates that the formation of the observed product is more complex than a single reaction occurring in competition with CH_2OO + $H_2O/(H_2O)_2$. The decrease in the observed product signal of 77 % between experiments performed in the absence of water vapour and those at the highest relative humidities is greater than the contribution of CH_2OO reactions with water vapour, which represented 22 % of the total loss of CH_2OO in the experiments performed at the highest RH (see Section S5.2). The extent of the change in the observed product signal indicates that the product

formation involves multiple steps, several of which occur in competition with reactions involving water. We speculate that the absorption results from species generated from chemistry of the SOZ or HMF formed via CH_2OO + HCHO, or oligomer formation resulting from CH_2OO + HCOOH. Previous work has indicated that Criegee intermediates can undergo insertion reactions with carboxylic acids to form hydroperoxide esters, leading to formation of oligomers (*72*) and, ultimately, secondary organic aerosol, with several studies of CH_2OO chemistry also demonstrating the potential for oligomer formation.(*73-75*)

6. Ozonolysis of tetramethyl ethene (TME)

6.1 Initial conditions

Table S4 summarises the initial conditions used in experiments to investigate the ozonolysis of TME. Experiments were performed at total pressures of 100 and 1000 mbar, and with initial concentrations ranging from excess O_3 to excess TME in order to provide sensitivity to pressure-dependent SCI yields and reaction kinetics.

6.2 Model analysis

The ozonolysis of TME was investigated using the same model framework described for the ozonolysis of ethene, which was developed in the ChemPy and SciPy packages in Python and represented mixing in HIRAC as described in Section S4. The mechanism used in the model is given in Table S5 and summarised in Figure S10.

In contrast to the model used to describe the ozonolysis of ethene, the model did include OH radical chemistry in order to account for OH-initiated losses of TME which have the potential to impact the determination of the SCI yield for experiments in which O_3 was in excess over TME. The rate coefficients for O_3 + TME and OH + TME were constrained to current IUPAC recommendations.(*42*) Decomposition of the excited Criegee intermediate and SCI were assumed to produce OH and a peroxy radical, $CH_3C(O)CH_2O_2$, in unity yield,(*9*) with HCHO produced following subsequent chemistry of the peroxy radical. Production of HCHO was represented as a first-order process in the model although it strictly results from second-order chemistry of the peroxy radical involving self-reaction and other species. The model included production of acetone, CH_3COCH_3, in the initial ozonolysis reaction, with unity yield, and from OH + TME with a yield that was varied to fit to the acetone observations made by FT-IR spectroscopy. Further production from chemistry of the SCI was not included owing to uncertainties in reaction yields, although production from SCI chemistry is likely and yields of acetone resulting from OH + TME are thus likely overestimates but enable the model to reproduce the acetone concentrations.

Modelled concentrations of $(CH_3)_2COO$, HCHO, acetone, and O_3 were fit globally to observations made by CEAS, for $(CH_3)_2COO$ and HCHO, and FT-IR, for acetone and O_3, for all experiments listed in Table S4 to investigate SCI yields and kinetics of SCI decomposition and bimolecular reactions. The model was initialised with concentrations of ozone and TME. Fits to observations of $(CH_3)_2COO$, HCHO, acetone, and O_3 from all experiments listed in Table S4 were made simultaneously, with the fit parameters treated globally and shared between all datasets. Observations and modelled concentrations were weighted in the same manner as for modelling of the ethene system (Section S5) in order to avoid potential biases in the fits owing to differences between the concentrations of different species of several orders of magnitude.

The fit results are summarised in Table S5, with example fits shown in Figure S11. The best fit to the observations gave SCI yields of (0.45 ± 0.09) at 100 mbar and (0.61 ± 0.18) at 1000 mbar and pressure-independent rate coefficients for decomposition and reaction with HCHO of (156 ± 68) s^{-1} and $(9.7 \pm 6.8) \times 10^{-13}$ cm^3 s^{-1}, respectively. Potential impacts of SCI self-reaction and reactions with acetone, O_3, and TME were also investigated, but were not found to significantly improve the fit quality. Further details are given in Section S6.5.

Figure S12 shows the relative contributions of the SCI reactions in the model to the total SCI loss, using the results for the SCI yield and kinetics determined in this work, for the mean initial concentrations of O_3 and TME used at 1000 mbar. Decomposition of stabilised $(CH_3)_2COO$ was the dominant SCI removal process in the model, representing 88 % of the total SCI loss, while reaction with HCHO represented 12 % of the total SCI loss.

6.3 SCI Yields

Table S6 summarises previous measurements of SCI yields in the ozonolysis of TME, which have indicated pressure-dependent yields but range between 0.1 and 0.65 at atmospheric pressure. Results obtained in this work indicate a yield of (0.45 ± 0.09) at 100 mbar and (0.61 ± 0.18) at 1000 mbar.

Pressure measurements of SCI yields have typically involved the use of SCI scavengers such as SO_2 or hexafluoroacetone that are expected to react with the SCI to generate products such as H_2SO_4 or secondary ozonides that can be quantified relative to the consumption of reactants. In some cases, calibration of product signals is required, which can lead to significant uncertainties in SCI yields. Calibration uncertainties of up to 45 % have been reported for H_2SO_4 measurements used to determine the SCI yield from TME ozonolysis.(*59, 76*) Use of scavengers also requires that sufficient scavenger concentrations are used to compete effectively with other SCI reactions, with incomplete competition leading to determination of the minimum yield,(*58*) whilst potential losses of the SCI-scavenger product, to aerosol for example, also limit the yield determined.(*38*)

6.4 SCI Decomposition Kinetics

Results from this work indicate a decomposition rate coefficient of (156 ± 68) s^{-1}, with no significant dependence on pressure between 100 and 1000 mbar. Table S7 summarises previous studies of stabilised $(CH_3)_2COO$ decomposition kinetics at ~298 K. While there are significant discrepancies between results reported in previous work, several studies have been performed over a range of pressures and are in agreement that the kinetics are independent of pressure over the ranges investigated. The lack of pressure dependence has been attributed to the enhancement of the reaction rate resulting from quantum tunnelling, which dominates the overall rate and possible effects of pressure.(*60-62*)

Indirect measurements, made by observations of OH produced during the ozonolysis of TME have been used to estimate SCI decomposition rate coefficients of (2.7 ± 0.7) s^{-1} at 10 Torr and (6.4 ± 0.9) s^{-1} at 100 Torr.(*24*) The indirect measurements (*24*) provided the first reports of the SCI thermal decomposition kinetics but are potentially impacted by losses of OH on the timescale of the measurements, which would limit the apparent production rate of OH used to determine the SCI kinetics. Relative rate measurements (*25, 59, 76*) made using ozonolysis of TME and observations of H_2SO_4 formation or SO_2 removal resulting from the reaction of $(CH_3)_2COO$ with SO_2 in competition with decomposition are significantly higher than the indirect measurements based on observations of OH production. The relative rate measurements, using the current IUPAC recommended kinetics for $(CH_3)_2COO + SO_2$,(*9*) and assuming no other losses of the SCI apart from decomposition or reaction with SO_2, have led to reports of decomposition rate coefficients between (605 ± 109) s^{-1} (*59*) and (929 ± 220) s^{-1}.(*25*)

Absolute measurements have been made using photolytic precursors to $(CH_3)_2COO$.(*21-23, 26*) However, there are differences between studies using photolysis of 1,1-diiodopropane ($(CH_3)_2CI_2$), which have reported rate coefficients at 298 K between (305 ± 70) s^{-1} (*23*) and (370 ± 34) s^{-1},(*21*) and a study using photolysis of 1-bromo-1-iodopropane ($(CH_3)_2CIBr$),(*26*) which reported a rate coefficient of (899 ± 42) s^{-1} at 296 K. While these studies considered potential impacts of Criegee-Criegee and Criegee-radical chemistry, there is the possibility of secondary chemistry that leads to more rapid SCI removal than expected. The 1-bromo-1-iodopropane precursor has the potential to generate Br atoms and BrO radicals, as well as I atoms and IO radicals and other species generated

by the 1,1-diiodopropane precursor, and is thus potentially more susceptible to impacts of secondary chemistry which may explain the differences in results obtained between studies using the different precursors. Differences between the absolute measurements using the diiodo precursor and the relative rate measurements may arise owing to incomplete consideration of SCI losses, such as self-reaction or reaction with HCHO, in the relative rate measurements could lead to overestimation of the decomposition kinetics.

The current IUPAC recommendation (*9*) for the rate coefficient for decomposition of $(CH_3)_2COO$ at 298 K is $(400\,^{+234}_{-148})$ s^{-1}, with no expected impact of pressure. The rate coefficient for decomposition of (156 ± 68) s^{-1} determined in this work is lower than previous absolute measurements and relative rate determinations, but is the first to be determined by direct observations of the SCI in an ozonolysis reaction.

6.5 SCI Bimolecular Reaction Kinetics

The kinetics for reaction of $(CH_3)_2COO$ with HCHO have been determined in this work for the first time, giving a rate coefficient of $(9.7 \pm 6.8) \times 10^{-13}$ cm^3 s^{-1}. Inclusion of SCI reactions with O_3 and/or acetone did not significantly improve the fit quality and gave poorly defined rate coefficients on the order of 10^{-14}-10^{-13} cm^3 s^{-1} for reaction with O_3 and 10^{-13} cm^3 s^{-1} for reaction with acetone. Inclusion of a reaction between the SCI and TME resulted in significantly poorer fits to the SCI. For model runs which included reactions of the SCI with O_3, acetone, and/or TME as well as decomposition and reaction with HCHO, the fraction of SCI loss to reactions other than decomposition or that with HCHO was negligible.

A rapid self-reaction of $(CH_3)_2COO$ has been indicated in laser flash photolysis studies using diiodo precursors, with reports of $(1.6 \pm 1.3) \times 10^{-10}$ cm^3 s^{-1} (*77*) and $(6.0 \pm 1.1) \times 10^{-10}$ cm^3 s^{-1}.(*23*) However, the self-reaction kinetics reported in the photolytic studies are significantly higher than those determined for the Criegee intermediate CH_2OO, which has a recommended (*9*) rate coefficient of $(7.4^{+1.9}_{-1.5}) \times 10^{-11}$ cm^3 s^{-1}. The photolytic studies may have been impacted by reactions of the Criegee intermediate $(CH_3)_2COO$ with iodine atoms and/or iodine monoxide radicals, which have been shown to impact determinations of the self-reaction kinetics for the Criegee intermediate CH_2OO. Inclusion of SCI self-reaction in the model did not significantly improve the fit quality.

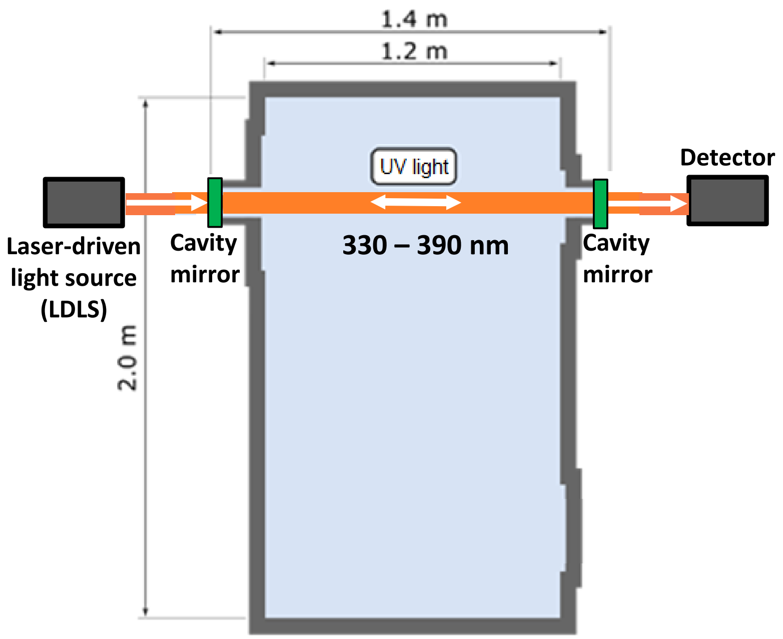

Figure S1: Schematic of the Highly Instrumented Reactor for Atmospheric Chemistry (HIRAC).

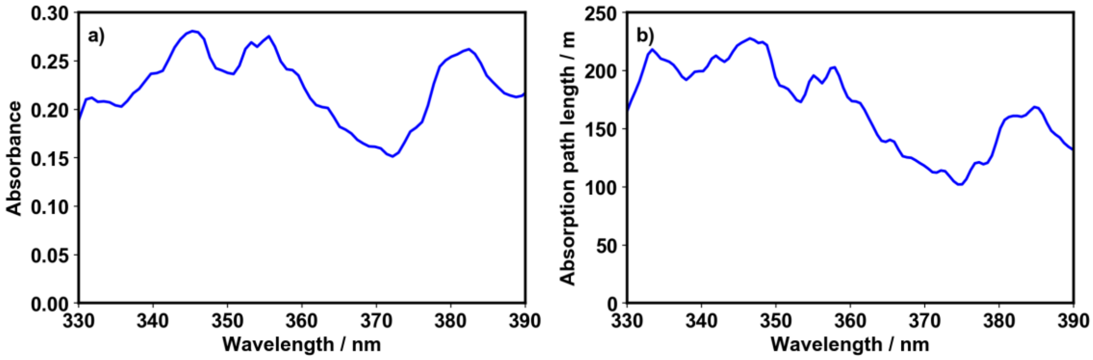

Figure S2: Determination of the CEAS path length. The plots show a) the measured absorbance by CEAS for a known NO_2 concentration of 7.1×10^{13} cm^{-3} in HIRAC used to determine the absorption path length as a function of wavelength, and b) the total absorption path length as a function of wavelength determined from the measured absorbance by NO_2 and reference absorption cross-sections for NO_2.(*42*)

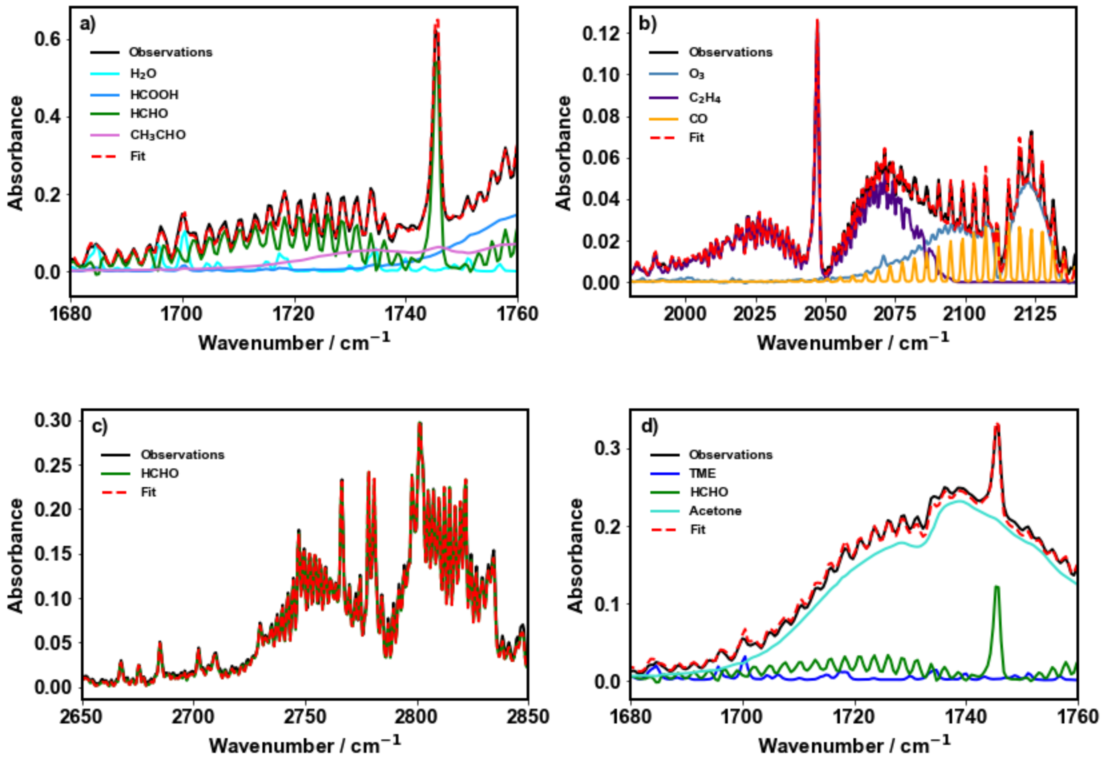

Figure S3: Example fits to FT-IR spectra. Experiments shown were initialised with a) $[C_2H_4] = 4.38 \times 10^{15}$ cm^{-3} and $[O_3] = 2.87 \times 10^{14}$ cm^{-3} 1000 mbar, b) $[C_2H_4] = 4.38 \times 10^{15}$ cm^{-3} and $[O_3] = 2.87 \times 10^{14}$ cm^{-3} at 1000 mbar, c) $[C_2H_4] = 4.38 \times 10^{15}$ cm^{-3} and $[O_3] = 2.87 \times 10^{14}$ cm^{-3} at 1000 mbar, and d) $[TME] = 1.45 \times 10^{14}$ cm^{-3} and $[O_3] = 7.07 \times 10^{13}$ cm^{-3}at 1000 mbar. Black solid lines show the observations, solid coloured lines show the contributions from each species in the fit, and the dashed red lines show the total fits to the observations.

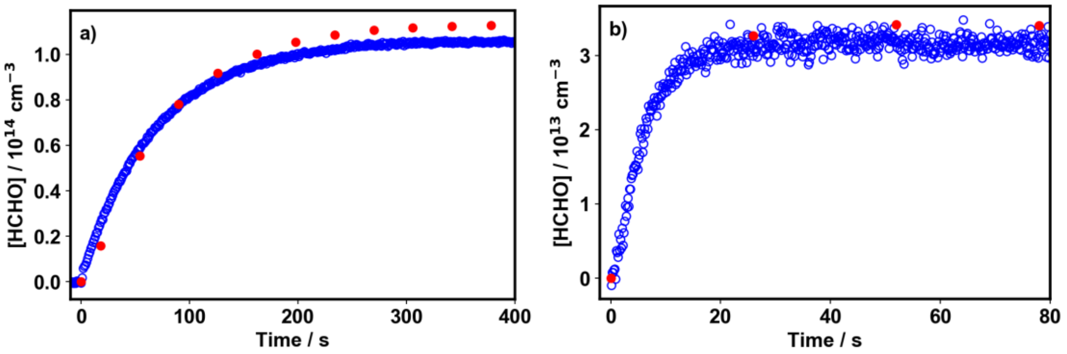

Figure S4: Comparison of HCHO concentration-time profiles determined from CEAS and FT-IR spectroscopy. Data show measurements by CEAS (blue) and FT-IR spectroscopy (red) for experiments initialised with a) [C_2H_4] = 9.26 × 10^{15} cm^{-3} and [O_3] = 1.36 × 10^{14} cm^{-3} at 1000 mbar, and b) [TME] = 2.32 × 10^{14} cm^{-3} and [O_3] = 6.24 × 10^{13} cm^{-3} at 100 mbar.

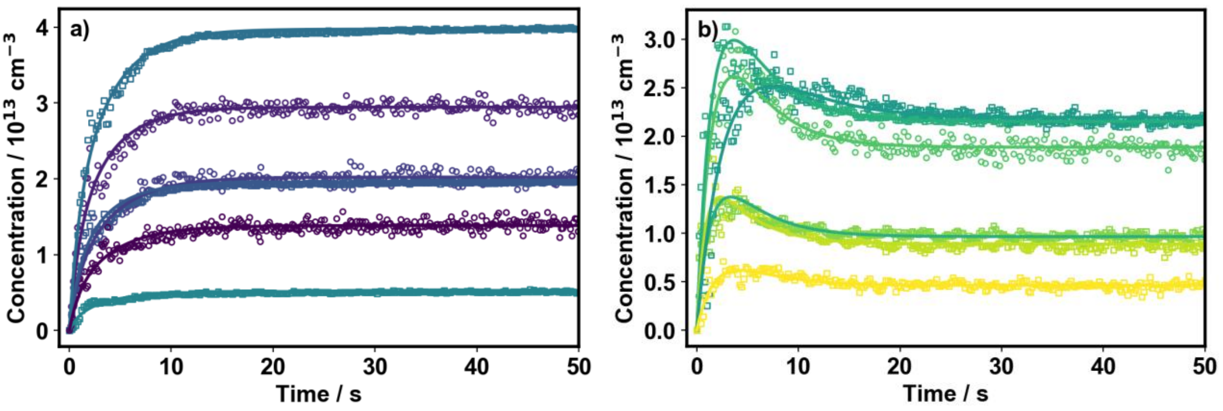

Figure S5: Concentration-time profiles used to determine mixing times in HIRAC. Profiles were obtained by delivering NO_2 (square symbols, red colours) and HCHO (circular symbols, blue colours) to HIRAC at 295 K and a) 100 mbar N_2 and b) 1000 mbar N_2. The experimental data were fit simultaneously at each pressure by Equation S2 (solid lines).

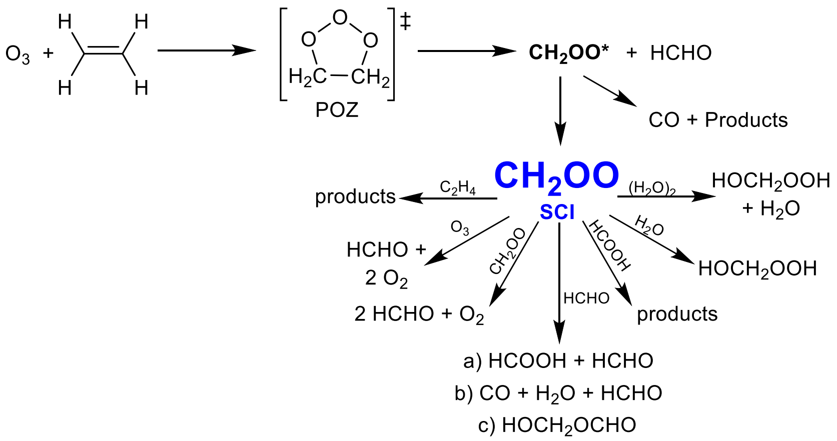

Figure S6: Mechanism used to describe the ozonolysis of ethene.

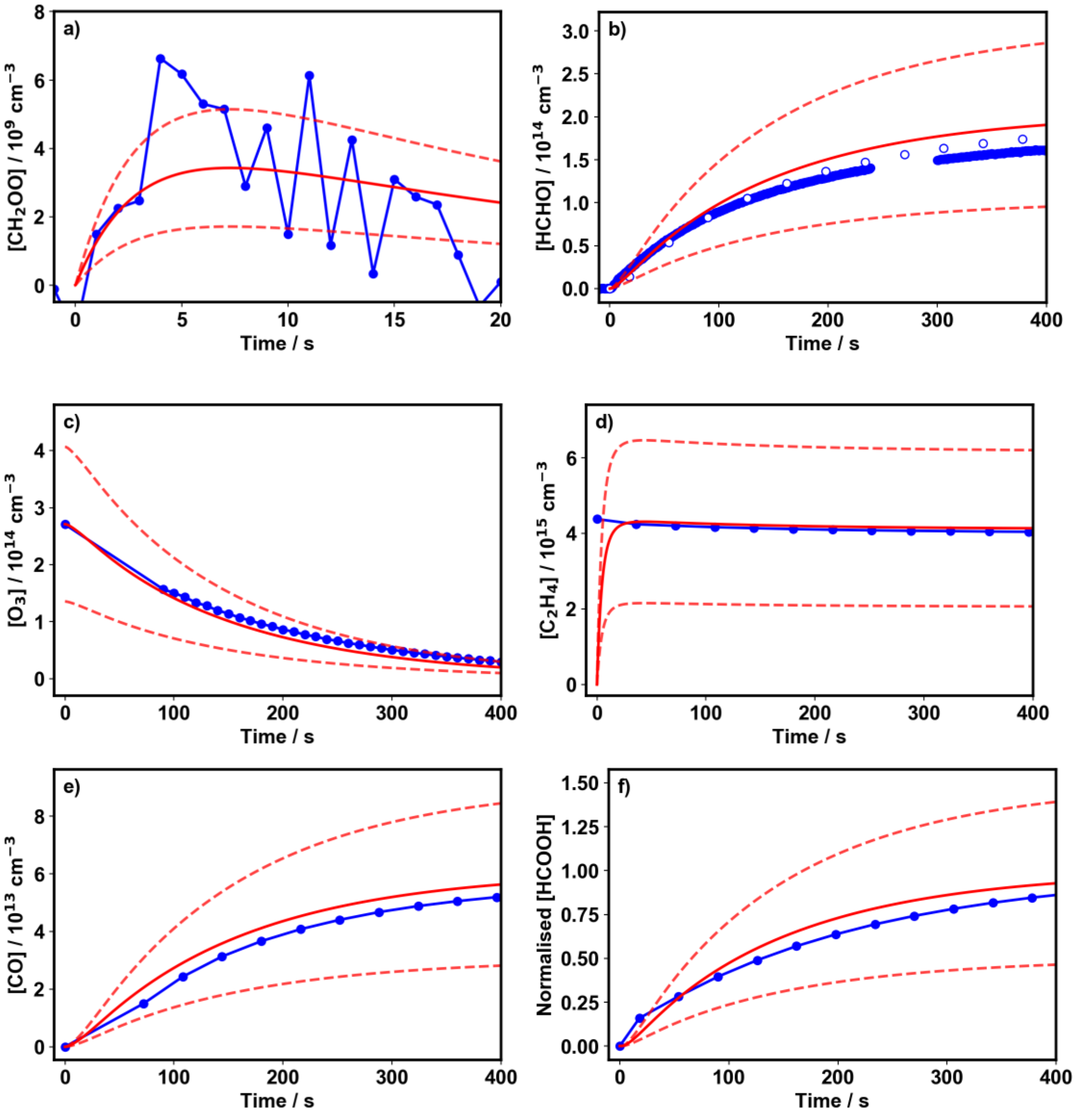

Figure S7: Observations and model fits for data obtained during the ozonolysis of ethene. Data show observed concentrations (blue) and model fits (red) for a) stabilised CH_2OO, b) HCHO (CEAS in filled symbols, FTIR in open symbols), c) O_3, d) C_2H_4, e) CO, and f) HCOOH (normalised to the maximum of the observations (blue) or modelled (red) concentration). Dashed lines show the modelled concentrations for each species increased or decreased by 50 %. Data shown are from an experiment initialised with 2.7×10^{14} cm^{-3} O_3 and 4.4×10^{15} cm^{-3} ethene, in the absence of water vapour.

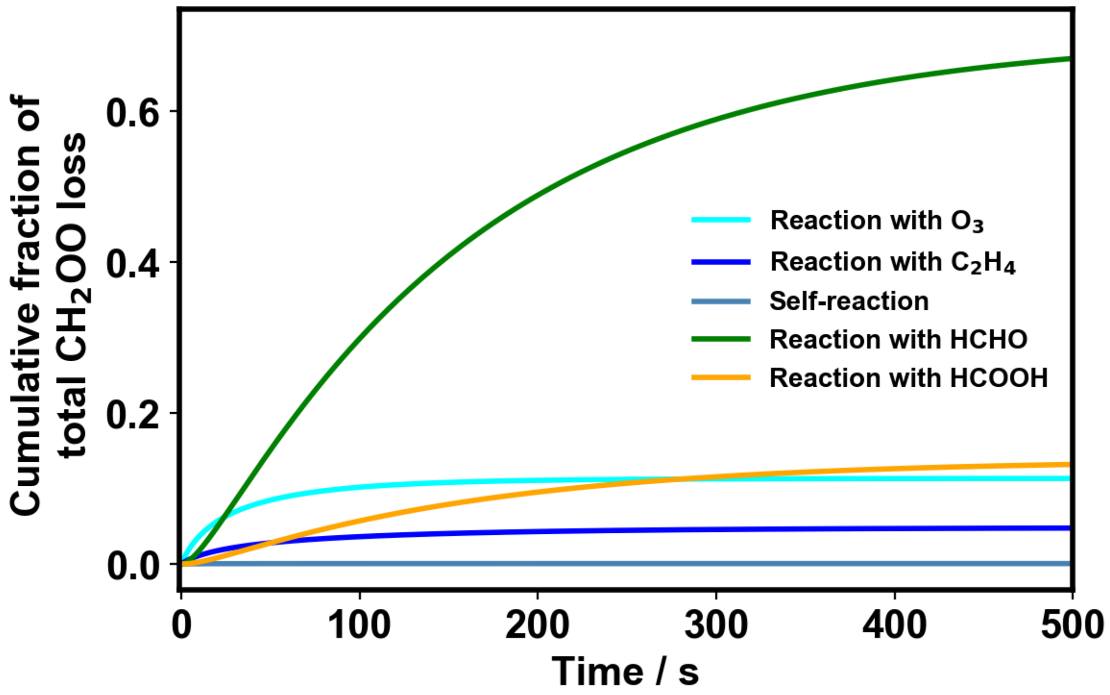

Figure S8: Relative cumulative contributions of the CH_2OO reactions in the model to the total CH_2OO loss. Results are shown from a simulation using the CH_2OO yield and kinetics determined in this work with $[C_2H_4] = 4.4 \times 10^{14}$ cm^{-3} and $[O_3] = 2.7 \times 10^{14}$ cm^{-3} in the absence of water vapour.

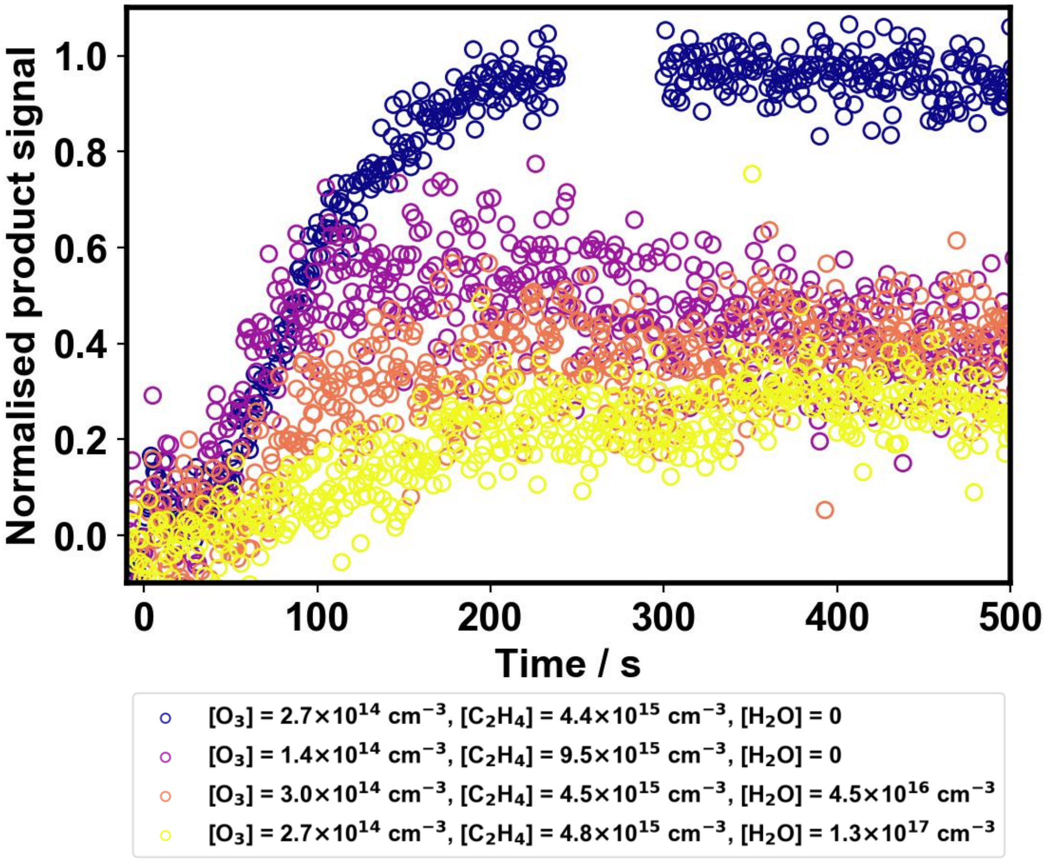

Figure S9: Time dependence of the unidentified product species observed by CEAS normalised to the maximum observed values. Data shown are from experiments with similar initial concentrations of O_3 and C_2H_4 for a range of relative humidities.

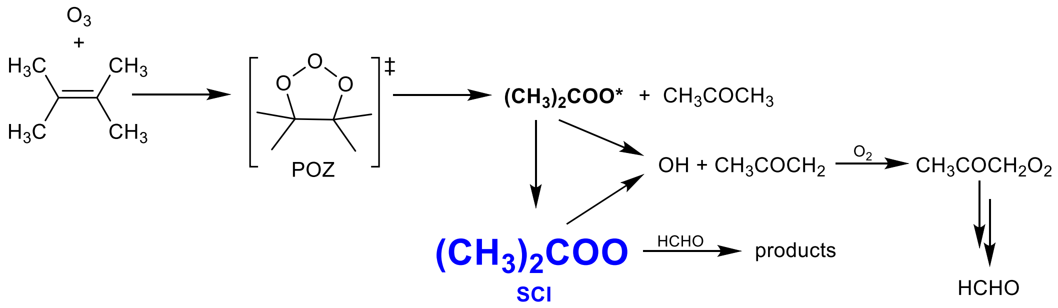

Figure S10: Mechanism used to describe the ozonolysis of TME.

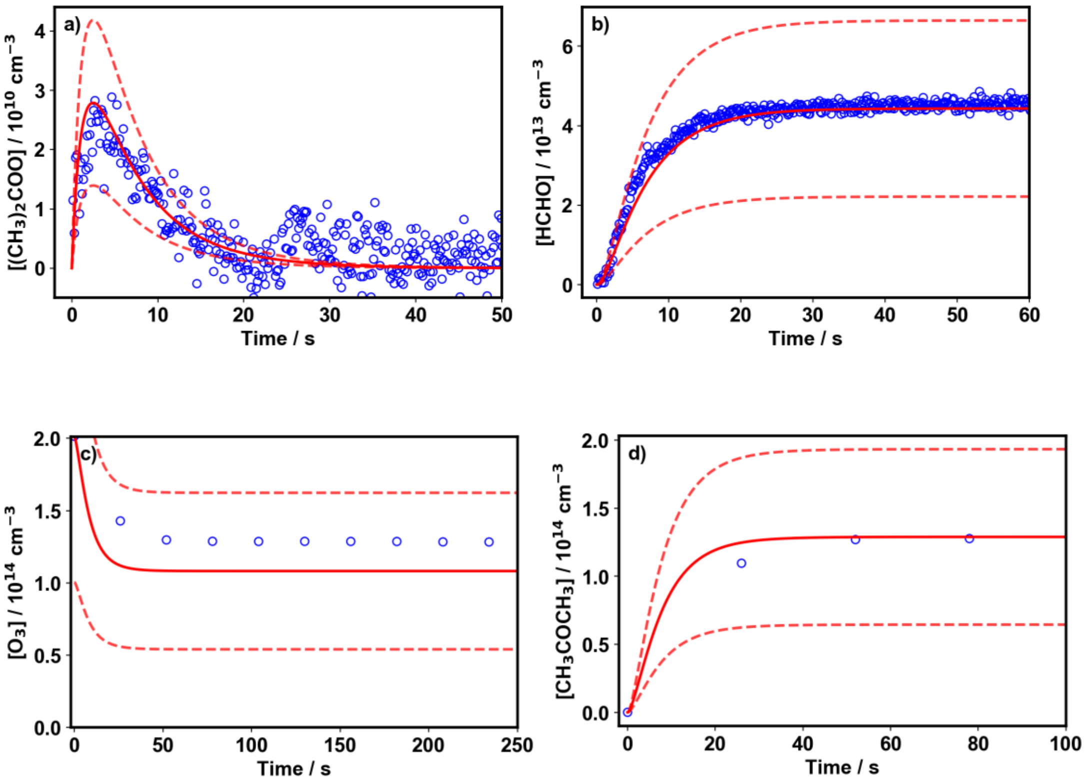

Figure S11: Observations and model fits for data obtained during the ozonolysis of TME. Data show observed concentrations (blue) and model fits (red) for a) stabilised $(CH_3)_2COO$, b) HCHO, c) O_3, and d) acetone. Dashed lines show the modelled concentrations for each species increased or decreased by 50 %. Data shown are from an experiment at 100 mbar initialised with 2.0×10^{14} cm^{-3} O_3 and 1.3×10^{14} cm^{-3} TME.

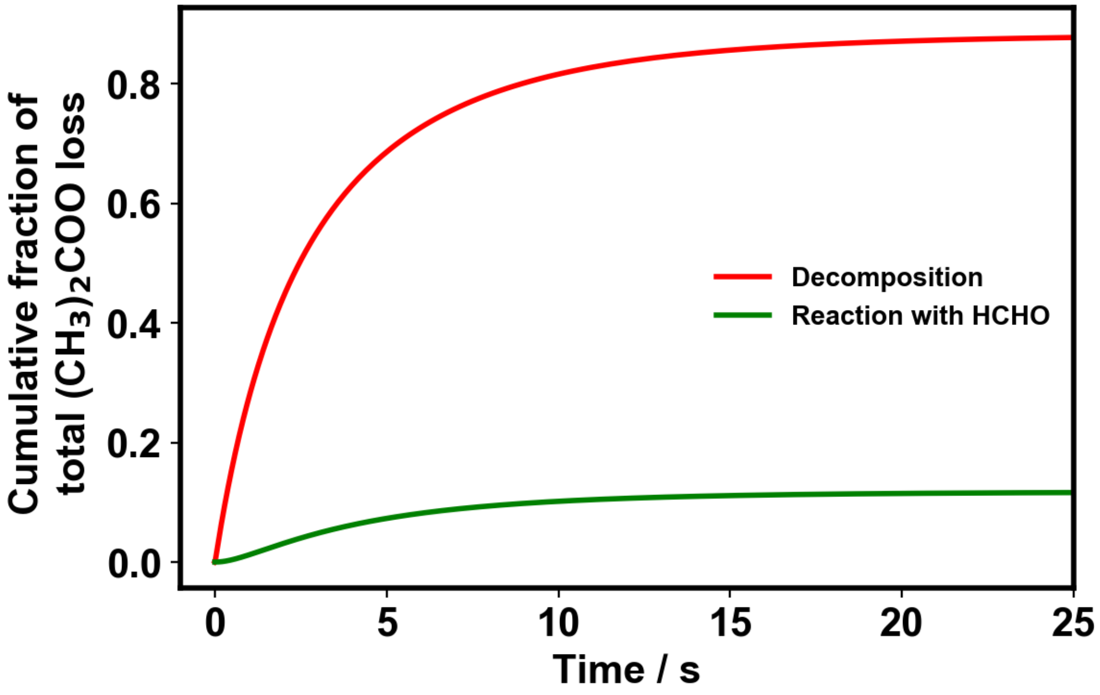

Figure S12: Relative cumulative contributions of the $(CH_3)_2COO$ reactions in the model to the total $(CH_3)_2COO$ loss. Results are shown from a simulation using the $(CH_3)_2COO$ yield and kinetics determined in this work with the mean initial concentrations of O_3 and TME used at 1000 mbar.

[Ozone] / 10^{14} cm^{-3}	[Ethene] / 10^{15} cm^{-3}	[H_2O] / 10^{16} cm^{-3}	[$(H_2O)_2$] / cm^{-3}
1.49	9.3	0	0
2.14	12.1	0	0
2.87	4.4	0	0
3.13	4.6	0.2	5.3×10^{9}
3.18	5.0	1.4	4.2×10^{11}
2.93	5.0	4.5	4.6×10^{12}
2.99	4.8	12.7	3.7×10^{13}

Table S1. Summary of initial conditions used in experiments to investigate the ozonolysis of ethene. All experiments to investigate the ozonolysis of ethene were performed at p = 1000 mbar and T = 295 K.

Reaction	Rate coefficient[a] / s^{-1} or $cm^3\ s^{-1}$	Reference
$C_2H_4 + O_3 \rightarrow y_{SCI}\ CH_2OO + y_{CO}\ CO + HCHO$	1.6×10^{-18}	IUPAC (*9*)
$CH_2OO + O_3 \rightarrow HCHO + 2\ O_2$	3.6×10^{-13}	Onel et al. (*78*)
$CH_2OO + C_2H_4 \rightarrow$ products	6.5×10^{-15}	Buras et al. (*79*)
$CH_2OO + CH_2OO \rightarrow 2\ HCHO + O_2$	8.0×10^{-11}	IUPAC (*9*)
$CH_2OO + HCHO \rightarrow y'_{CO}\ CO + y_{HCOOH}\ HCOOH + y_{HCHO}\ HCHO + (1\text{-}y_{HCHO})\ HOCH_2OCHO$	6.0×10^{-12}	This work
$CH_2OO + HCOOH \rightarrow$ products	1.1×10^{-10}	IUPAC (*9*)
$CH_2OO + H_2O \rightarrow HOCH_2OOH$	2.8×10^{-16}	IUPAC (*9*)
$CH_2OO + (H_2O)_2 \rightarrow HOCH_2OOH + H_2O$	6.4×10^{-12}	IUPAC (*9*)
$HCHO \rightarrow$ loss	10^{-5}	This work[b]

Table S2: Mechanism used in numerical simulations to fit to observations of CH_2OO, HCHO, O_3, and CO. The yields y_{SCI}, y_{CO}, y'_{CO}, and y_{HCHO} and the rate coefficient for reaction of CH_2OO with HCHO were determined from fits to observations. Yields of CO were determined to be $y_{CO} = 0.19$ and $y'_{CO} = 0.16$, but could not assigned independently with confidence (further details are given in Section S5.4). The yield y_{HCOOH} was fixed to the reported value of 0.23 unless otherwise stated (see Section S5.4). The reaction between CH_2OO and HCHO was assumed to produce HCOOH + HCHO, CO + H_2O + HCHO, or $HOCH_2OCHO$, with the yield of $HOCH_2OCHO$ (hydroxymethyl formate, HMF) thus given by ($1\text{-}y_{HCHO}$). [a] Units are $cm^3\ s^{-1}$ for second-order reactions and s^{-1} for first-order processes, [b] wall loss rates for HCHO were determined in this work in separate experiments to those in the presence of ozone and ethene.

Pressure	Yield	Method	Reference
700 Torr	0.38	HCHO, CH_3CHO, CO, and SO_2 scavengers	Su et al. (*50*)
700 Torr	0.35 ± 0.05	HCHO scavenger	Niki et al. (*32*)
700 Torr	0.37 ± 0.02	HCHO scavenger	Kan et al. (*51*)
1 atm	0.39 ± 0.05	SO_2 scavenger	Hatakeyama et al. (*80*)
10-1140 Torr[a]	0.4	SO_2 scavenger	Hatakeyama et al. (*52*)
758 Torr	0.47 ± 0.05	Total yield product yields and HCHO scavenger	Horie and Moortgat (*53*)
730 Torr	0.4-0.5	Total yield product yields and HCOOH, CH_3COOH, and CH_3OH scavengers	Neeb et al. (*81*)
730 Torr	0.50	Total yield product yields and HCHO and CH_3CHO scavengers	Neeb et al. (*82*)
730 Torr	0.52 ± 0.06	CF_3COCF_3 scavenger	Horie et al. (*83*)
1 atm	0.39 ± 0.11	H_2O and HCOOH scavengers	Hasson et al. (*34*)
~1 atm	0.54 ± 0.12	CO scavenger	Alam et al. (*54*)
1000 mbar	0.37 ± 0.04	SO_2 scavenger	Newland et al. (*25*)
1 atm	0.59 ± 0.17	CH_3COOH scavenger (dry conditions)	Yajima et al. (*33*)
1 atm	0.55 ± 0.16	CH_3COOH scavenger (humid conditions, RH 23-24 %)	Yajima et al. (*33*)
1000 mbar	0.43 ± 0.02	SO_2 scavenger	Newland et al. (*58*)
7-19 Torr[b]	0.214 ± 0.082 to 0.255 ± 0.033	SO_2 scavenger	Yang et al. (*39*)
4-19 Torr	0.23 ± 0.07 to 0.25 ± 0.07	Direct observation of CH_2OO	Campos-Pineda et al. (*31*)
1000 mbar	0.42 ± 0.10	Data evaluation	IUPAC (*9*)
1000 mbar	0.38 ± 0.09	Direct observation of CH_2OO	This work

Table S3: Summary of reported yields of stabilised CH_2OO from the ozonolysis of ethene. Detailed summaries and discussion of previous work are given by Alam et al.(*54*) and Newland et al.(*25*) [a] The SCI yield of 0.4 was reported by Hatakeyama et al.(*52*) for the high pressure limit, experiments were also performed at 10 Torr which indicated a lower, but non-zero, SCI yield. [b] Extrapolation to zero pressure gave a nascent SCI yield of (0.201 ± 0.025).(*39*)

p / mbar	[Ozone] / 10^{14} cm^{-3}	[TME] / 10^{14} cm^{-3}
100	0.6	1.1
100	0.6	2.3
100	1.2	1.5
100	1.9	1.0
100	2.0	1.3
100	3.0	0.8
100	4.3	0.5
1000	0.5	1.4
1000	0.8	3.1
1000	0.9	0.3
1000	1.2	3.4
1000	1.4	1.0
1000	1.8	1.1
1000	1.8	0.7
1000	3.4	0.6
1000	3.6	0.2
1000	7.1	1.5

Table S4. Summary of initial conditions used in experiments to investigate the ozonolysis of TME. All experiments to investigate the ozonolysis of TME were performed at $T = 295$ K.

Reaction	Rate coefficient[a] / s^{-1} or cm^{3} s^{-1}	Reference
O_3 + TME → y_{SCI} $(CH_3)_2COO$ + (1 - y_{SCI}) OH + (1 - y_{SCI}) $CH_3COCH_2O_2$ + CH_3COCH_3	1.1×10^{-15}	IUPAC(*9*)
$CH_3COCH_2O_2$ → y_{HCHO} HCHO	7[b], 45[c]	This work
$(CH_3)_2COO$ (+O_2) → $CH_3COCH_2O_2$ + OH	156	This work[d]
$(CH_3)_2COO$ + HCHO → loss	9.7×10^{-13}	This work
OH + TME → $y_{CH3COCH3}$ CH_3COCH_3	1.1×10^{-10}	IUPAC(*9*)
OH + HCHO → loss	8.5×10^{-12}	IUPAC(*9*)
OH + CH_3COCH_3 → loss	1.8×10^{-13}	IUPAC(*9*)
OH → loss	9[b], 13[c]	This work
HCHO → loss	10^{-5}	This work[e]

Table S5: Mechanism used in numerical simulations to fit to observations of $(CH_3)_2COO$, HCHO, O_3 and acetone. The yields y_{SCI}, y_{HCHO}, and $y_{CH3COCH3}$ were determined at 100 mbar and 1000 mbar from fits to this work. Rate coefficients for $(CH_3)_2COO$ decomposition and reaction with HCHO were determined in this work and were independent of pressure. The rate coefficients for production of HCHO from RO_2 and for losses of OH involving reactions other than those listed were treated as variables in the model. [a] Units are cm^{3} s^{-1} for second-order reactions and s^{-1} for first-order processes, [b] values determined at p = 100 mbar, [c] values determined at p = 1000 mbar, [d] The decomposition of $(CH_3)_2COO$ produces the radical CH_3COCH_2, which is assumed to undergo rapid reaction with O_2 under the conditions in the chamber to produce the peroxy radical $CH_3COCH_2O_2$, [e] wall loss rates for HCHO were determined in this work in separate experiments to those in the presence of ozone and TME.

**Pressure**	**Yield**	**Method**	**Reference**
700 Torr	~0.25	HCHO and CH_3CHO scavengers	Niki et al. (*84*)
730 Torr	0.29 ± 0.03	CF_3COCF_3 scavenger	Horie et al. (*83*)
760 Torr	0.11 ± 0.11	SO_2 scavenger	Rickard et al. (*85*)
760 Torr	0.1 ± 0.03	H_2O scavenger and detection of H_2O_2	Hasson et al. (*34*)
710 Torr	0.65 ± 0.2	CF_3COCF_3 scavenger	Drozd et al. (*35, 86*)
760 Torr	0.62 ± 0.28	SO_2 scavenger	Berndt et al. (*59*)
760 Torr	0.45 ± 0.2	SO_2 scavenger	Berndt et al. (*76*)
1000 mbar	0.32 ± 0.02	SO_2 scavenger	Newland et al. (*25*)
50 Torr	0.13 ± 0.06	SO_2 scavenger	Hakala and Donahue (*37*)
75 Torr	~0.14	SO_2 scavenger	Hakala and Donahue (*37*)
375 Torr	~0.25	SO_2 scavenger	Hakala and Donahue (*37*)
760 Torr	0.37 ± 0.01	SO_2 scavenger	Hakala and Donahue (*37*)
900 Torr	0.42 ± 0.02	SO_2 scavenger	Hakala and Donahue (*37*)
4-60 Torr[a]	0.14 ± 0.06 to ~0.3	SO_2 scavenger	Campos-Pineda and Zhang (*38*)
1000 mbar	0.31 ± 0.04	SO_2 scavenger	Newland et al. (*58*)
1000 mbar	0.38 ± 0.10	Data evaluation	IUPAC (*9*)
100 mbar	0.45 ± 0.09	Direct observation of $(CH_3)_2COO$	This work
1000 mbar	0.61 ± 0.18	Direct observation of $(CH_3)_2COO$	This work

Table S6: Summary of reported yields of stabilised $(CH_3)_2COO$ from the ozonolysis of TME. A detailed summary and discussion of previous work is given by Newland et al.(*25*) [a] Extrapolation to zero pressure gave a nascent SCI yield of (0.12 ± 0.05).(*38*)

T / K	*p*	*k* / s^{-1}	Method	Reference
298	10 Torr	2.7 ± 0.7	Indirect, via observation of OH production	Kroll et al. (*24*)
298	100 Torr	6.4 ± 0.9	Indirect, via observation of OH production	Kroll et al. (*24*)
293	760 Torr	605 ± 109 [a]	Relative rate	Berndt et al. (*59*)
293	760 Torr	722 ± 52 [a]	Relative rate	Berndt et al. (*76*)
298	1000 mbar	929 ± 220 [a]	Relative rate	Newland et al. (*25*)
298	200-500 Torr	370 ± 34	Absolute[b]	Huang et al. (*21*)
298	100-200 Torr	361 ± 49	Absolute[b]	Smith et al. (*22*)
298	10-100 Torr	305 ± 70	Absolute[b]	Chhantyal-Pun et al. (*23*)
296	200 Torr	899 ± 42	Absolute[c]	Peltola et al. (*26*)
298	-	400^{+234}_{-148}	Data evaluation	IUPAC (*9*)
295	100-1000 mbar	156 ± 68	Absolute[d]	This work

Table S7: Summary of experimental determinations of rate coefficients for stabilised $(CH_3)_2COO$ decomposition. [a] Results of relative rate studies have been updated to the most recent IUPAC recommendations (*9*) for kinetics of $(CH_3)_2COO + SO_2$, which was used as the reference reaction. [b] Absolute measurements reported by Huang et al.,(*21*) Smith et al.,(*22*) and Chhantyal-Pun et al.(*23*) used photolysis of 1,1-diiodopropane ($(CH_3)_2CI_2$) to generate $(CH_3)_2COO$. [c] Absolute measurements reported by Peltola et al.(*26*) used photolysis of 1-bromo-1-iodopropane ($(CH_3)_2CIBr$) to generate $(CH_3)_2COO$. [d] Absolute measurements reported in this work used O_3 + TME to generate $(CH_3)_2COO$.

Data S1: Data used to generate the main figures presented in this study are available in the Supplementary Data File.

REFERENCES

1. D. Johnson, G. Marston, The gas-phase ozonolysis of unsaturated volatile organic compounds in the troposphere. *Chem. Soc. Rev.* **37**, 699–716 (2008).

2. N. M. Donahue, G. T. Drozd, S. A. Epstein, A. A. Presto, J. H. Kroll, Adventures in ozoneland: Down the rabbit-hole. *Phys. Chem. Chem. Phys.* **13**, 10848–10857 (2011).

3. C. J. Percival, O. Welz, A. J. Eskola, J. D. Savee, D. L. Osborn, D. O. Topping, D. Lowe, S. R. Utembe, A. Bacak, G. McFiggans, M. C. Cooke, P. Xiao, A. T. Archibald, M. E. Jenkin, R. G. Derwent, I. Riipinen, D. W. K. Mok, E. P. F. Lee, J. M. Dyke, C. A. Taatjes, D. E. Shallcross, Regional and global impacts of Criegee intermediates on atmospheric sulphuric acid concentrations and first steps of aerosol formation. *Faraday Discuss.* **165**, 45–73 (2013).

4. C. A. Taatjes, D. E. Shallcross, C. J. Percival, Research frontiers in the chemistry of Criegee intermediates and tropospheric ozonolysis. *Phys. Chem. Chem. Phys.* **16**, 1704–1718 (2014).

5. M. J. Newland, A. R. Rickard, T. Sherwen, M. J. Evans, L. Vereecken, A. Muñoz, M. Ródenas, W. J. Bloss, The atmospheric impacts of monoterpene ozonolysis on global stabilised Criegee intermediate budgets and SO_2 oxidation: Experiment, theory and modelling. *Atmos. Chem. Phys.* **18**, 6095–6120 (2018).

6. M. A. H. Khan, C. J. Percival, R. L. Caravan, C. A. Taatjes, D. E. Shallcross, Criegee intermediates and their impacts on the troposphere. *Environ. Sci. Process. Impacts* **20**, 437–453 (2018).

7. D. L. Osborn, C. A. Taatjes, The physical chemistry of Criegee intermediates in the gas phase. *Int. Rev. Phys. Chem.* **34**, 309–360 (2015).

8. C. A. Taatjes, in *Annual Review of Physical Chemistry*, *Vol 68*, M. A. Johnson, T. J. Martinez, Eds. (2017), vol. 68, pp. 183–207.

9. R. A. Cox, M. Ammann, J. N. Crowley, H. Herrmann, M. E. Jenkin, V. F. McNeill, A. Mellouki, J. Troe, T. J. Wallington, Evaluated kinetic and photochemical data for atmospheric chemistry: Volume VII - Criegee intermediates. *Atmos. Chem. Phys.* **20**, 13497–13519 (2020).

10. R. Criegee, G. Wenner, Die Ozonisierung Des 9,10-Oktalins. *Justus Liebigs Ann. Chem.* **564**, 9–15 (1949).

11. R. A. Cox, S. A. Penkett, Photo-oxidation of atmospheric SO_2. *Nature* **229**, 486-& (1971), 488.

12. R. A. Cox, S. A. Penkett, Oxidation of atmospheric SO_2 by products of the ozone-olefin reaction. *Nature* **230**, 321-& (1971), 322.

13. R. A. Cox, S. A. Penkett, Effect of relative humidity on the disappearance of ozone and sulphur dioxide in contained systems. *Atmos. Environ.* **6**, 365–368(1972).

14. R. A. Cox, S. A. Penkett, Aerosol formation from sulphur dioxide in the presence of ozone and olefinic hydrocarbons. *J. Chem. Soc. Faraday Trans.* **68**, 1735 (1972).

15. D. H. F. Atkins, R. A. Cox, A. E. Eggleton, Photochemical ozone and sulphuric acid aerosol formation in the atmosphere over Southern England. *Nature* **235**, 372–376 (1972).

16. C. A. Taatjes, G. Meloni, T. M. Selby, A. J. Trevitt, D. L. Osborn, C. J. Percival, D. E. Shallcross, Direct observation of the gas-phase Criegee intermediate (CH_2OO). *J. Am. Chem. Soc.* **130**, 11883–11885 (2008).

17. O. Welz, J. D. Savee, D. L. Osborn, S. S. Vasu, C. J. Percival, D. E. Shallcross, C. A. Taatjes, Direct kinetic measurements of Criegee intermediate (CH_2OO) formed by reaction of Ch2i with O2. *Science* **335**, 204–207 (2012).

18. C. A. Taatjes, O. Welz, A. J. Eskola, J. D. Savee, A. M. Scheer, D. E. Shallcross, B. Rotavera, E. P. F. Lee, J. M. Dyke, D. K. W. Mok, D. L. Osborn, C. J. Percival, Direct measurements of conformer-dependent reactivity of the Criegee intermediate CH_3CHOO. *Science* **340**, 177–180 (2013).

19. R. Chhantyal-Pun, M. A. H. Khan, C. A. Taatjes, C. J. Percival, A. J. Orr-Ewing, D. E. Shallcross, Criegee intermediates: Production, detection and reactivity. *Int. Rev. Phys. Chem.* **39**, 383–422 (2020).

20. R. L. Caravan, M. F. Vansco, M. I. Lester, Open questions on the reactivity of Criegee intermediates. *Commun. Chem.* **4**, 44 (2021).

21. H. L. Huang, W. Chao, J. J. M. Lin, Kinetics of a Criegee intermediate that would survive high humidity and may oxidize atmospheric SO_2. *Proc. Natl. Acad. Sci. U.S.A.* **112**, 10857–10862 (2015).

22. M. C. Smith, W. Chao, K. Takahashi, K. A. Boering, J. J. M. Lin, Unimolecular decomposition rate of the Criegee intermediate $(CH_3)_2COO$ measured directly with UV absorption spectroscopy. *J. Phys. Chem. A* **120**, 4789–4798 (2016).

23. R. Chhantyal-Pun, O. Welz, J. D. Savee, A. J. Eskola, E. P. F. Lee, L. Blacker, H. R. Hill, M. Ashcroft, M. A. H. Khan, G. C. Lloyd-Jones, L. Evans, B. Rotavera, H. F. Huang, D. L. Osborn, D. K. W. Mok, J. M. Dyke, D. E. Shallcross, C. J. Percival, A. J. Orr-Ewing, C. A. Taatjes, Direct measurements of unimolecular and bimolecular reaction kinetics of the Criegee intermediate $(CH_3)_2COO$. *J. Phys. Chem. A* **121**, 4–15 (2017).

24. J. H. Kroll, S. R. Sahay, J. G. Anderson, K. L. Demerjian, N. M. Donahue, Mechanism of HO_x formation in the gas-phase ozone-alkene reaction.: 2.: Prompt versus thermal dissociation of carbonyl oxides to form OH. *J. Phys. Chem. A* **105**, 4446–4457 (2001).

25. M. J. Newland, A. R. Rickard, M. S. Alam, L. Vereecken, A. Muñoz, M. Ródenas, W. J. Bloss, Kinetics of stabilised Criegee intermediates derived from alkene ozonolysis: Reactions with SO_2, H_2O and decomposition under boundary layer conditions. *Phys. Chem. Chem. Phys.* **17**, 4076–4088 (2015).

26. J. Peltola, P. Seal, N. Vuorio, P. Heinonen, A. Eskola, Solving the discrepancy between the direct and relative-rate determinations of unimolecular reaction kinetics of dimethyl-substituted

Criegee intermediate $(CH_3)_2COO$ using a new photolytic precursor. *Phys. Chem. Chem. Phys.* **24**, 5211–5219 (2022).

27. C. C. Womack, M. A. Martin-Drumel, G. G. Brown, R. W. Field, M. C. McCarthy, Observation of the simplest Criegee intermediate CH_2OO in the gas-phase ozonolysis of ethylene. *Sci. Adv.* **1**, e1400105 (2015).

28. J. Ahrens, P. T. M. Carlsson, N. Hertl, M. Olzmann, M. Pfeifle, J. L. Wolf, T. Zeuch, Infrared detection of Criegee intermediates formed during the ozonolysis of β-pinene and their reactivity towards sulfur dioxide. *Angew. Chem. Int. Ed Engl.* **53**, 715–719 (2014).

29. C. Giorio, S. J. Campbell, M. Bruschi, F. Tampiere, A. Barbon, A. Toffoletti, A. Tapparo, C. Paijens, A. J. Wedlake, P. Grice, D. J. Howe, M. Kalberer, Online quantification of Criegee intermediates of α-pinene ozonolysis by stabilization with spin traps and proton-transfer reaction mass spectrometry Detection. *J. Am. Chem. Soc.* **139**, 3999–4008 (2017).

30. C. Giorio, S. J. Campbell, M. Bruschi, A. T. Archibald, M. Kalberer, Detection and identification of Criegee intermediates from the ozonolysis of biogenic and anthropogenic VOCs: Comparison between experimental measurements and theoretical calculations. *Faraday Discuss.* **200**, 559–578 (2017).

31. M. Campos-Pineda, L. Yang, J. S. Zhang, Direct measurement of the Criegee intermediate CH_2OO in ozonolysis of ethene. *Nat. Commun.* **16**, 6515 (2025).

32. H. Niki, P. D. Maker, C. M. Savage, L. P. Breitenbach, A FT-IR study of a transitory product in the gas-phase ozone-ethylene reaction. *J. Phys. Chem.* **85**, 1024–1027 (1981).

33. R. Yajima, Y. Sakamoto, S. Inomata, J. Hirokawa, Relative reactivity measurements of stabilized CH_2OO, produced by ethene ozonolysis, toward acetic acid and water vapor using chemical ionization mass spectrometry. *J. Phys. Chem. A* **121**, 6440–6449 (2017).

34. A. S. Hasson, G. Orzechowska, S. E. Paulson, Production of stabilized Criegee intermediates and peroxides in the gas phase ozonolysis of alkenes 1.: Ethene, trans-2-butene, and 2,3-dimethyl-2-butene. *J. Geophys. Res. Atmos.* **106**, 34131–34142 (2001).

35. G. T. Drozd, N. M. Donahue, Pressure dependence of stabilized Criegee intermediate formation from a sequence of alkenes. *J. Phys. Chem. A* **115**, 4381–4387 (2011).

36. S. M. Saunders, M. E. Jenkin, R. G. Derwent, M. J. Pilling, Protocol for the development of the master chemical mechanism, MCM V3 (Part a): Tropospheric degradation of non-aromatic volatile organic compounds. *Atmos. Chem. Phys.* **3**, 161–180 (2003).

37. J. P. Hakala, N. M. Donahue, Pressure-dependent Criegee intermediate stabilization from alkene ozonolysis. *J. Phys. Chem. A* **120**, 2173–2178 (2016).

38. M. Campos-Pineda, J. S. Zhang, Low-pressure yields of stabilized Criegee intermediates CH_3CHOO and $(CH_3)_2COO$ in ozonolysis of trans-2-butene and 2,3-dimethyl-2-butene. *Chem. Phys. Lett.* **683**, 647–652 (2017).

39. L. Yang, M. Campos-Pineda, J. S. Zhang, Low-pressure and nascent yields of thermalized Criegee intermediate in ozonolysis of ethene. *J. Phys. Chem. Lett.* **13**, 11496–11502 (2022).

40. D. R. Glowacki, A. Goddard, K. Hemavibool, T. L. Malkin, R. Commane, F. Anderson, W. J. Bloss, D. E. Heard, T. Ingham, M. J. Pilling, P. W. Seakins, Design of and initial results from a Highly Instrumented Reactor for Atmospheric Chemistry (HIRAC). *Atmos. Chem. Phys.* **7**, 5371–5390 (2007).

41. Z. S. Mir, T. R. Lewis, L. Onel, M. A. Blitz, P. W. Seakins, D. Stone, CH_2OO Criegee intermediate UV absorption cross-sections and kinetics of CH_2OO + CH_2OO and CH_2OO + I as a function of pressure. *Phys. Chem. Chem. Phys.* **22**, 9448–9459 (2020).

42. R. Atkinson, D. L. Baulch, R. A. Cox, J. N. Crowley, R. F. Hampson, R. G. Hynes, M. E. Jenkin, M. J. Rossi, J. Troe, IUPAC Subcommittee, Evaluated kinetic and photochemical data for atmospheric chemistry: Volume II – Gas phase reactions of organic species. *Atmos. Chem. Phys.* **6**, 3625–4055 (2006).

43. W. Chao, J. T. Hsieh, C. H. Chang, J. J. M. Lin, Direct kinetic measurement of the reaction of the simplest Criegee intermediate with water vapor. *Science* **347**, 751–754 (2015).

44. R. E. Lade, M. A. Blitz, M. Rowlinson, M. J. Evans, P. W. Seakins, D. Stone, Kinetics of the reactions of the Criegee intermediate CH_2OO with water vapour: Experimental measurements as a function of temperature and global atmospheric modelling. *Environ. Sci. Atmos.* **4**, 1294–1308 (2024).

45. P. L. Luo, I. Y. Chen, M. A. H. Khan, D. E. Shallcross, Direct gas-phase formation of formic acid through reaction of Criegee intermediates with formaldehyde. *Commun. Chem.* **6**, 130 (2023).

46. J. J. Enders, Z. A. Cornwell, A. W. Harrison, C. Murray, Temperature-dependent kinetics of the reactions of the Criegee intermediate CH_2OO with aliphatic aldehydes. *J. Phys. Chem. A* **128**, 7879–7888 (2024).

47. A. Jalan, J. W. Allen, W. H. Green, Chemically activated formation of organic acids in reactions of the Criegee intermediate with aldehydes and ketones. *Phys. Chem. Chem. Phys.* **15**, 16841–16852 (2013).

48. C. Elakiya, R. Shankar, S. Vijayakumar, P. Kolandaivel, A theoretical study on the reaction mechanism and kinetics of allyl alcohol (CH_2=$CHCH_2OH$) with ozone (O_3) in the atmosphere. *Mol. Phys.* **115**, 895–911 (2017).

49. B. Long, Y. Wang, Y. Xia, X. He, J. L. Bao, D. G. Truhlar, Atmospheric kinetics: Bimolecular reactions of carbonyl oxide by a triple-level strategy. *J. Am. Chem. Soc.* **143**, 8402–8413 (2021).

50. F. Su, J. G. Calvert, J. H. Shaw, FT-IR spectroscopic study of the ozone ethene reaction-mechanism in O_2-rich mixtures. *J. Phys. Chem.* **84**, 239–246 (1980).

51. C. S. Kan, F. Su, J. G. Calvert, J. H. Shaw, Mechanism of the ozone-ethene reaction in dilute N_2-O_2 mixtures near 1-atm pressure. *J. Phys. Chem.* **85**, 2359–2363 (1981).

52. S. Hatakeyama, H. Kobayashi, Z. Y. Lin, H. Takagi, H. Akimoto, Mechanism for the reaction of CH_2OO with SO_2. *J. Phys. Chem.* **90**, 4131–4135 (1986).

53. O. Horie, G. K. Moortgat, Decomposition pathways of the excited Criegee intermediates in the ozonolysis of simple alkenes. *Atmos. Environ. Part A Gen. Top.* **25**, 1881–1896 (1991).

54. M. S. Alam, M. Camredon, A. R. Rickard, T. Carr, K. P. Wyche, K. E. Hornsby, P. S. Monks, W. J. Bloss, Total radical yields from tropospheric ethene ozonolysis. *Phys. Chem. Chem. Phys.* **13**, 11002–11015 (2011).

55. O. Welz, A. J. Eskola, L. Sheps, B. Rotavera, J. D. Savee, A. M. Scheer, D. L. Osborn, D. Lowe, A. M. Booth, P. Xiao, M. A. H. Khan, C. J. Percival, D. E. Shallcross, C. A. Taatjes, Rate coefficients of C_1 and C_2 Criegee intermediate reactions with formic and acetic acid near the collision limit: Direct kinetics measurements and atmospheric implications. *Angew. Chem. Int. Ed Engl.* **53**, 4547–4550 (2014).

56. R. Chhantyal-Pun, B. Rotavera, M. R. McGillen, M. A. H. Khan, A. J. Eskola, R. L. Caravan, L. Blacker, D. P. Tew, D. L. Osborn, C. J. Percival, C. A. Taatjes, D. E. Shallcross, A. J. Orr-Ewing, Criegee intermediate reactions with carboxylic acids: A potential source of secondary organic aerosol in the atmosphere. *ACS Earth Space Chem.* **2**, 833–842 (2018).

57. J. Peltola, P. Seal, A. Inkilä, A. Eskola, Time-resolved, broadband UV-absorption spectrometry measurements of Criegee intermediate kinetics using a new photolytic precursor: Unimolecular decomposition of CH_2OO and its reaction with formic acid. *Phys. Chem. Chem. Phys.* **22**, 11797–11808 (2020).

58. M. J. Newland, B. S. Nelson, A. Muñoz, M. Ródenas, T. Vera, J. Tárrega, A. R. Rickard, Trends in stabilisation of Criegee intermediates from alkene ozonolysis. *Phys. Chem. Chem. Phys.* **22**, 13698–13706 (2020).

59. T. Berndt, T. Jokinen, R. L. Mauldin, T. Petäjä, H. Herrmann, H. Junninen, P. Paasonen, D. R. Worsnop, M. Sipilä, Gas-phase ozonolysis of selected olefins: The yield of stabilized Criegee intermediate and the reactivity toward SO_2. *J. Phys. Chem. Lett.* **3**, 2892–2896 (2012).

60. Y. Fang, V. P. Barber, S. J. Klippenstein, A. B. McCoy, M. I. Lester, Tunneling effects in the unimolecular decay of $(CH_3)_2COO$ Criegee intermediates to OH radical products. *J. Chem. Phys.* **146**, 134307 (2017).

61. L. Vereecken, A. Novelli, D. Taraborrelli, Unimolecular decay strongly limits the atmospheric impact of Criegee intermediates. *Phys. Chem. Chem. Phys.* **19**, 31599–31612 (2017).

62. M. I. Lester, S. J. Klippenstein, Unimolecular decay of Criegee intermediates to OH radical products: prompt and thermal decay processes. *Acc. Chem. Res.* **51**, 978–985 (2018).

63. S. Srsen, D. Hollas, P. Slavícek, UV absorption of Criegee intermediates: Quantitative cross sections from high-level ab initio theory. *Phys. Chem. Chem. Phys.* **20**, 6421–6430 (2018).

64. J. C. McCoy, B. Marchetti, M. Thodika, T. N. Karsili, A simple and efficient method for simulating the electronic absorption spectra of Criegee intermediates: benchmarking on CH_2OO and CH_3CHOO. *J. Phys. Chem. A* **125**, 4089–4097 (2021).

65. J. C. McCoy, S. J. Léger, C. F. Frey, M. F. Vansco, B. Marchetti, T. N. Karsili, Modeling the conformer-dependent electronic absorption spectra and photolysis rates of methyl vinyl ketone oxide and methacrolein oxide. *J. Phys. Chem. A* **126**, 485–496 (2022).

66. K. Takahashi, Wave packet calculation of absolute UV cross section of Criegee intermediates. *J. Phys. Chem. A* **126**, 6080–6090 (2022).

67. Y. P. Chang, C. H. Chang, K. Takahashi, J. J. M. Lin, Absolute UV absorption cross sections of dimethyl substituted Criegee intermediate $(CH_3)_2COO$. *Chem. Phys. Lett.* **653**, 155–160 (2016).

68. D. R. Glowacki, A. Goddard, P. W. Seakins, Design and performance of a throughput-matched, zero-geometric-loss, modified three objective multipass matrix system for FTIR spectrometry. *Appl. Optics* **46**, 7872–7883 (2007).

69. W. L. Ting, Y. H. Chen, W. Chao, M. C. Smith, J. J. M. Lin, The UV absorption spectrum of the simplest Criegee intermediate CH_2OO. *Phys. Chem. Chem. Phys.* **16**, 10438–10443 (2014).

70. E. S. Foreman, K. M. Kapnas, Y. Jou, J. Kalinowski, D. Feng, R. B. Gerber, C. Murray, High resolution absolute absorption cross sections of the B^1A'-X^1A' transition of the CH_2OO biradical. *Phys. Chem. Chem. Phys.* **17**, 32539–32546 (2015).

71. D. Stone, K. Au, S. Sime, D. J. Medeiros, M. Blitz, P. W. Seakins, Z. Decker, L. Sheps, Unimolecular decomposition kinetics of the stabilised Criegee intermediates CH_2OO and CD_2OO. *Phys. Chem. Chem. Phys.* **20**, 24940–24954 (2018).

72. L. Chen, Y. Huang, Y. G. Xue, Z. H. Jia, W. L. Wang, Oligomer formation from the gas-phase reactions of Criegee intermediates with hydroperoxide esters: Mechanism and kinetics. *Atmos. Chem. Phys.* **22**, 14529–14546 (2022).

73. Y. Sakamoto, S. Inomata, J. Hirokawa, Oligomerization reaction of the Criegee intermediate leads to secondary organic aerosol formation in ethylene ozonolysis. *J. Phys. Chem. A* **117**, 12912–12921 (2013).

74. Y. Zhao, L. M. Wingen, V. Perraud, J. Greaves, B. J. Finlayson-Pitts, Role of the reaction of stabilized Criegee intermediates with peroxy radicals in particle formation and growth in air. *Phys. Chem. Chem. Phys.* **17**, 12500–12514 (2015).

75. R. L. Caravan, T. J. Bannan, F. A. F. Winiberg, M. A. H. Khan, A. C. Rousso, A. W. Jasper, S. D. Worrall, A. Bacak, P. Artaxo, J. Brito, M. Priestley, J. D. Allan, H. Coe, Y. Ju, D. L. Osborn, N. Hansen, S. J. Klippenstein, D. E. Shallcross, C. A. Taatjes, C. J. Percival, Observational evidence for Criegee intermediate oligomerization reactions relevant to aerosol formation in the troposphere. *Nat. Geosci.* **17**, 219, 226 (2024).

76. T. Berndt, T. Jokinen, M. Sipila, R. L. Mauldin, H. Herrmann, F. Stratmann, H. Junninen, M. Kulmala, H_2SO_4 formation from the gas-phase reaction of stabilized Criegee intermediates with SO_2: Influence of water vapour content and temperature. *Atmos. Environ.* **89**, 603–612 (2014).

77. Y.-Y. Wang, C.-Y. Chung, Y.-P. Lee, Infrared spectral identification of the Criegee intermediate $(CH_3)_2COO$. *J. Chem. Phys.* **145**, 154303 (2016).

78. L. Onel, M. Blitz, P. Seakins, D. Heard, D. Stone, Kinetics of the gas phase reactions of the Criegee intermediate CH_2OO with O_3 and IO. *J. Phys. Chem. A* **124**, 6287–6293 (2020).

79. Z. J. Buras, R. M. I. Elsamra, A. Jalan, J. E. Middaugh, W. H. Green, Direct kinetic measurements of reactions between the simplest Criegee intermediate CH_2OO and alkenes. *J. Phys. Chem. A* **118**, 1997–2006 (2014).

80. S. Hatakeyama, H. Kobayashi, H. Akimoto, Gas-phase oxidation of SO_2 in the ozone olefin reactions. *J. Phys. Chem.* **88**, 4736–4739 (1984).

81. P. Neeb, O. Horie, G. K. Moortgat, Gas-phase ozonolysis of ethene in the presence of hydroxylic compounds. *Int. J. Chem. Kinet.* **28**, 721–730 (1996).

82. P. Neeb, O. Horie, G. K. Moortgat, The ethene-ozone reaction in the gas phase. *J. Phys. Chem. A* **102**, 6778–6785 (1998).

83. O. Horie, C. Schäfer, G. K. Moortgat, High reactivity of hexafluoro acetone toward Criegee intermediates in the gas-phase ozonolysis of simple alkenes. *Int. J. Chem. Kinet.* **31**, 261–269 (1999).

84. H. Niki, P. D. Maker, C. M. Savage, L. P. Breitenbach, M. D. Hurley, FTIR spectroscopic study of the mechanism for the gas-phase reaction between ozone and tetramethylethylene. *J. Phys. Chem.* **91**, 941–946 (1987).

85. A. R. Rickard, D. Johnson, C. D. McGill, G. Marston, Oh yields in the gas-phase reactions of ozone with alkenes. *J. Phys. Chem. A* **103**, 7656–7664 (1999).

86. G. T. Drozd, J. Kroll, N. M. Donahue, 2,3-Dimethyl-2-butene (TME) ozonolysis: Pressure dependence of stabilized Criegee intermediates and evidence of stabilized vinyl hydroperoxides. *J. Phys. Chem. A* **115**, 161–166 (2011).
